# ATAXIC: An Algorithm to Quantify Transcriptomic Perturbation Heterogeneity in Single Cancer Cells

**DOI:** 10.1155/2022/4106736

**Published:** 2022-08-31

**Authors:** Qian Liu, Qiqi Lu, Xiaosheng Wang

**Affiliations:** ^1^Biomedical Informatics Research Lab, School of Basic Medicine and Clinical Pharmacy, China Pharmaceutical University, Nanjing 211198, China; ^2^Cancer Genomics Research Center, School of Basic Medicine and Clinical Pharmacy, China Pharmaceutical University, Nanjing 211198, China; ^3^Big Data Research Institute, China Pharmaceutical University, Nanjing 211198, China

## Abstract

The single-cell RNA sequencing (scRNA-seq) has recently been widely utilized to quantify transcriptomic profiles in single cells of bulk tumors. The transcriptomic profiles in single cells facilitate the investigation of intratumor heterogeneity that is unlikely confounded by the nontumor components. We proposed an algorithm (ATAXIC) to quantify the heterogeneity of transcriptomic perturbations (TPs) in single cancer cells. ATAXIC calculated the TP heterogeneity level of a single cell based on the standard deviations of the absolute z-scored gene expression values for tens of thousands of genes, reflecting the asynchronous degree of transcriptomic alterations relative to the central (mean) tendency. By analyzing scRNA-seq datasets for eight cancer types, we revealed that ATAXIC scores were likely to correlate positively with the enrichment scores of various proliferation and oncogenic signatures, DNA damage repair, treatment resistance, and unfavorable phenotypes and outcomes in cancer. The ATAXIC scores varied among different cancer types, with lung cancer and melanoma having the lowest average scores and clear cell renal cell carcinoma having the highest average scores. The low TP heterogeneity in lung cancer and melanoma could bestow relatively higher response rates to immune checkpoint inhibitors on both cancer types. In conclusion, ATAXIC is a useful algorithm to quantify the TP heterogeneity in single cancer cells, as well as providing new insights into tumor biology.

## 1. Introduction

Single-cell technology is rapidly evolving to analyze molecular characteristics at the single cell level [1]. In particular, the single-cell RNA sequencing (scRNA-seq) has shown its power in analyzing gene expression profiles in thousands of individual cells in one test [2, 3]. A major advantage of the scRNA-seq technology is that it enables characterization of sophisticated biological processes at single-cell resolution to capture transcriptional snapshots of rare events [4, 5]. Thus, this technology has recently been widely used to quantify transcriptomic profiles in different components of bulk tumors, including cancer cells, immune cells, stromal cells, and normal cells [6–13]. Apparently, scRNA-seq demonstrates the advantage of uncovering transcriptomic heterogeneity among different tumor cells as compared to bulk RNA sequencing, which measures the average expression of genes among different types of cells [14]. Therefore, scRNA-seq has evolved into a popular tool to dissect cellular heterogeneity in an unbiased manner without requiring any prior information of the cell population [2, 15, 16]. Most of the scRNA-seqdata-based studies aimed to characterize the tumor microenvironment, the cross-talk among the different components of bulk tumors, and intratumor heterogeneity (ITH). Previous studies on bulk tumors have demonstrated that the transcriptomic perturbations (TPs) are observed in a wide variety of cancers and are heterogeneous within a tumor and across different tumors [17, 18]. In fact, the heterogeneity of TPs is an intrinsic feature of many cancers and is due to the presence of different subclones within one tumor, each of which has its own unique features of gene expression profiles [19, 20]. Because the heterogeneity of TPs provides the fuel for drug resistance, a precise measure of ITH resulting from it is essential for the development of effective therapies [21]. However, the investigation into the TP profiles in single cancer cells remains lacking to date.

In this study, we developed an algorithm (ATAXIC) to quantify the TP heterogeneity in single cancer cells. The TP heterogeneity score indicates the asynchronous degree of transcriptomic alterations relative to the central tendency in single cells. Using ATAXIC, we scored the TP heterogeneity for single cells from eight cancer types, including glioma, melanoma, lung cancer, prostate cancer, renal cell carcinoma (RCC), liver cancer, breast cancer, and sarcoma. By analyzing correlations of ATAXIC scores with various oncogenic signaling, clinical features, DNA damage repair signatures, and treatment responses in these cancer types, we demonstrated that the ATAXIC index likely increases with tumor proliferation, progression, invasion, and metastasis and thus is a risk factor for tumor development.

## 2. Materials and Methods

### 2.1. Data Collection and Preprocess

We obtained the data of scRNA-seq gene expression profiles in single cells for eight caner types from the Gene Expression Omnibus (GEO) (https://www.ncbi.nlm.nih.gov/geo/), including glioma [13], melanoma [12], lung cancer [10], prostate cancer [8], RCC [6], liver cancer [9], breast cancer [7], and sarcoma (https://www.ncbi.nlm.nih.gov/geo/query/acc.cgi?acc=GSE131309). We excluded the patients with less than ten cancer cells reported in this study. Low-quality cells were discarded if the number of expressed genes was smaller than 300. Cells were also removed if their mitochondrial gene expression was larger than 10 percent. The numbers of patients and their cancer single cells for each cancer type are presented in Supplementary Table S1. We normalized all TPM normalized gene expression values by adding 1 and then log2 transformation. In addition, we downloaded the data of drug sensitivity of 578 cancer cell lines to 4686 compounds from the Dependency Map (DepMap) (https://depmap.org/portal/download/). We obtained the marker genes of proliferation, differentiation, invasion, metastasis, stemness, and damage repair signatures from the CancerSEA database (http://biocc.hrbmu.edu.cn/CancerSEA/home.jsp). We downloaded the pathway genes for the TGF-*β*, Wnt, PI3K-Akt, JAK-STAT, Notch, Hedgehog, mismatch repair, and homologous recombination pathways from KEGG [22]. The marker or pathway gene sets are given in Supplementary Table S2.

### 2.2. ATAXIC Algorithm

Given a normalized single cell gene expression matrix, which contains *m* genes and *t* samples (single cancer cells), the ATAXIC score of a single cell *SC* is defined as

(1) $$\begin{array}{c} \sqrt{\frac{\sum_{i=1}^{m}{\left(z\left(ex\left(G_{i},SC\right)\right)-\left(1/m\right)\sum_{i=1}^{m}z\left(ex\left(G_{i},SC\right)\right)\right)}^{2}}{m-1}}\\ =\sqrt{\frac{\sum_{i=1}^{m}{\left(\begin{array}{c} \left(\left|ex\left(G_{i},SC\right)-\left(1/t\right)\sum_{j=1}^{t}ex\left(G_{i},SS_{j}\right)\right|/\sqrt{\left({\sum_{j=1}^{t}\left(ex\left(G_{i},SC\right)-\left(1/t\right)\sum_{j=1}^{t}ex\left(G_{i},SS_{j}\right)\right)}^{2}/t-1\right)}\right)-\\ \left(1/m\right)\sum_{i=1}^{m}\left(\left|ex\left(G_{i},SC\right)-\left(1/t\right)\sum_{j=1}^{t}ex\left(G_{i},SS_{j}\right)\right|/\sqrt{\left(\sum_{j=1}^{t}{\left(ex\left(G_{i},SC\right)-\left(1/t\right)\sum_{j=1}^{t}ex\left(G_{i},SS_{j}\right)\right)}^{2}/t-1\right)}\right) \end{array}\right)}^{2}}{m-1}}\\ z\left(ex\left(G_{i},SC\right)\right)=\frac{\left|ex\left(G_{i},SC\right)-\left(1/t\right)\sum_{j=1}^{t}ex\left(G_{i},SS_{j}\right)\right|}{SD_{i}},\\ SD_{i}=\sqrt{\frac{\sum_{j=1}^{t}{\left(ex\left(G_{i},SC\right)-\left(1/t\right)\sum_{j=1}^{t}ex\left(G_{i},SS_{j}\right)\right)}^{2}}{t-1}}\text{,} \end{array}$$

where *ex* (*G*_*i*_, *SC*) denotes gene *G*_*i*_ expression level in *SC* and *ex* (*G*_*i*_, *SS*_*j*_) denotes *G*_*i*_ expression level in the single cell sample *SS*_*j*_.

**Figure 1:**
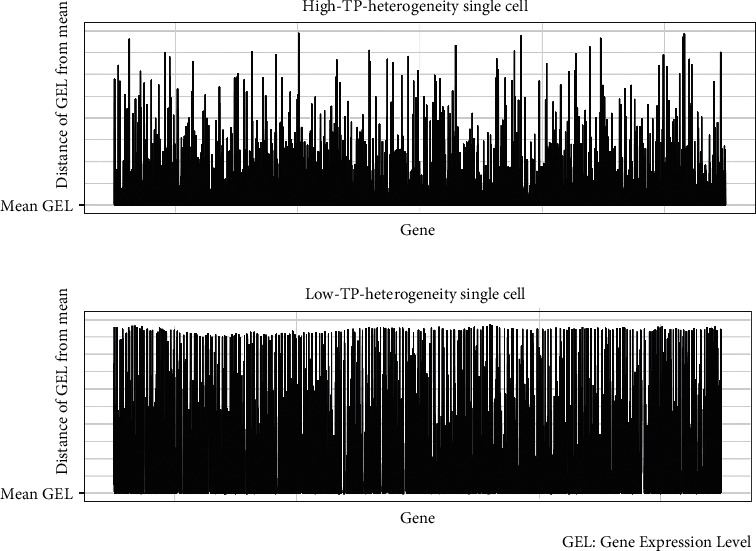
ATAXIC scores showing the asynchronous degree of transcriptomic alterations relative to the central (mean) tendency in single cancer cells.

ATAXIC calculated the TP heterogeneity level of a single cell based on the standard deviations of the absolute z-scored gene expression values for tens of thousands of genes. In a single cancer cell, when most genes show close absolute z-scored expression values, the single cancer cell will have a low ATAXIC score, namely, low TP heterogeneity level; otherwise, the single cancer cell will have a relatively high ATAXIC score. Hence, the ATAXIC score reflects the asynchronous degree of transcriptomic alterations relative to the central (mean) tendency normalized by standard deviation in all single cancer cells for all genes in the gene expression matrix (Figure 1). We present the *R* function for the ATAXIC algorithm at the GitHub (https://github.com/XS-Wang-Lab/ATAXIC/) under a GNU GPL open-source license.

### 2.3. Enrichment Scores of Molecular Signature or Pathways

For a molecular signature or pathway, we defined its enrichment score in a single cell as the average expression level of its marker or pathway genes in the single cell.

### 2.4. Correlations of ATAXIC Scores with Drug Sensitivity

We assessed the Spearman correlation of ATAXIC scores with viability values to each of the 4686 compounds in 578 cancer cell lines. The ATAXIC scores of cancer cell lines were calculated based on their gene expression profiles. The significant correlations were identified using a threshold of false discovery rate (FDR) < 0.05.

### 2.5. Statistical Analysis

Because ATAXIC scores did not follow the Gaussian distribution in most cases (Shapiro test, *P* < 0.05), we used the one-tailed Mann–Whitney *U* test to compare ATAXIC scores between two classes of samples. We evaluated correlations between ATAXIC scores and other variables using the Spearman method and reported the correlation coefficient (*ρ*) and adjusted *P* values. The adjusted *P* values were the FDRs calculated by the Benjamini and Hochberg method [23]. We performed all the statistical analyses in the R programming environment (version 4.0.2).

## 3. Results

### 3.1. ATAXIC Scores Likely Correlate Positively with Proliferation and Differentiation Signatures in *Cancer*

**Figure 2:**
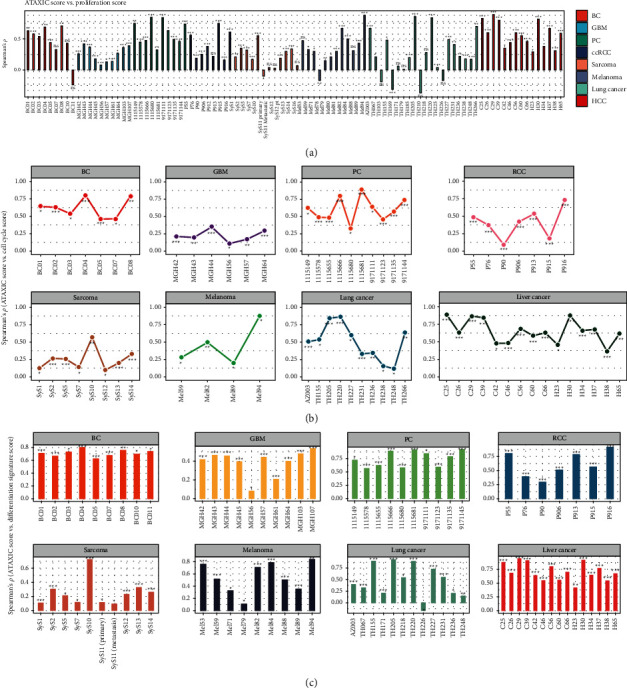
Correlations of ATAXIC scores with the proliferation and differentiation signatures in cancer. Spearman correlations between ATAXIC scores and the enrichment scores of the proliferation signature (a), the cell cycle pathway (b), and the differentiation signature (c) in single cells from individual patients of eight cancer types. BC: breast cancer; GBM: gliomas; PC: prostate cancer; RCC: renal cell carcinoma; SyS12 (pt): SyS12 post treatment. The Spearman correlation coefficients (p) and adjusted *P* values (FDR) are shown. ^∗^ FDR < 0.05, ^∗∗^ FDR < 0.01, ^∗∗∗^ FDR < 0.001, ns: not significant (this also applies to the following figures).

We defined the proliferation signature score in a single cell as the average expression levels of its marker genes [24]. We found that ATAXIC scores had a significant positive correlation with proliferation signature scores in 5 of the 9 breast cancers, 9 of the 10 gliomas, 9 of the 10 prostate cancers, 7 of the 8 RCCs, 8 of the 12 sarcomas, 7 of the 12 melanomas, 10 of the 20 lung cancers, and 13 of the 15 liver cancers (Spearman correlation, FDR < 0.05) (Figure 2(a)). Only in 1 sarcoma and 2 lung cancers, ATAXIC scores had a significant negative correlation with proliferation signature scores. The cell cycle is a process of cell growth and division. We found that ATAXIC scores were significantly and positively correlated with the enrichment scores of the cell cycle pathway in 7 breast cancers, 6 gliomas, 10 prostate cancers, 7 RCCs, 8 sarcomas, 4 melanomas, 10 lung cancers, and all the 15 liver cancers (Figure 2(b)). Only in 1 sarcoma, 2 melanomas, and 2 lung cancers, ATAXIC scores had a significant negative correlation with cell cycle scores. In addition, ATAXIC scores had a significant positive correlation with cell differentiation signature scores in most of these cancers, including 9 breast cancers, 10 gliomas, 10 prostate cancers, 7 RCCs, 10 sarcomas, 9 melanomas, 11 lung cancers, and 15 liver cancers (Figure 2(c)). Together, these results indicate that ATAXIC scores likely correlate positively with proliferation and differentiation potential in cancer.

### 3.2. ATAXIC Scores Likely Correlate Positively with Invasion, Metastasis, and Stemness Signatures in *Cancer*

**Figure 3:**
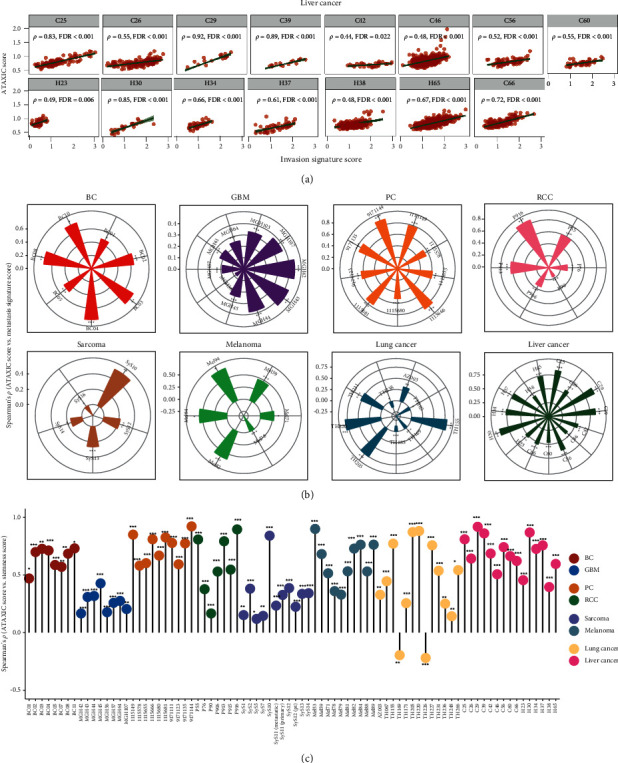
Correlations of ATAXIC scores with the invasion, metastasis, and stemness signatures in cancer. (a) Spearman correlations between ATAXIC scores and the enrichment scores of the invasion signature in single cells from liver cancer. Spearman correlations between ATAXIC scores and the enrichment scores of the metastasis (b) and stemness signatures (c) in single cells from individual patients of eight cancer types. The Spearman correlation coefficients and adjusted *P* values (FDR) are shown.

We analyzed correlations of ATAXIC scores with aggressive phenotypes in single cancer cells, including invasion, metastasis, and stemness signatures. We found that ATAXIC scores had a significant positive correlation with invasion signature scores in 6 breast cancers, 5 gliomas, 9 prostate cancers, 6 RCCs, 4 sarcomas, 5 melanomas, 6 lung cancers, and 15 liver cancers (Spearman correlation, FDR < 0.05) (Figure 3(a) and Supplementary Figure S1), compared to the significant negative correlation between them in 1 sarcoma, 1 melanoma, and 4 lung cancers. Moreover, ATAXIC scores were significantly and positively correlated with metastasis signature scores in 7 breast cancers, 10 gliomas, 9 prostate cancers, 6 RCCs, 4 sarcomas, 5 melanomas, 5 lung cancers, and 15 liver cancers (Spearman correlation, FDR < 0.05) (Figure 3(b)), compared to the significant negative correlation between them in 1 sarcoma, 1 melanoma, and 4 lung cancers. In addition, ATAXIC scores had a significant positive correlation with stemness signature scores in 8 breast cancers, 8 gliomas, 10 prostate cancers, 7 RCCs, 11 sarcomas, 10 melanomas, 11 lung cancers, and 15 liver cancers (Spearman correlation, FDR < 0.05) (Figure 3(c)), compared to the significant negative correlation with stemness signature scores ATAXIC scores display in 2 lung cancers. Collectively, these results indicate that ATAXIC scores likely have a positive correlation with the aggressive phenotypes in cancer.

### 3.3. ATAXIC Scores Likely Correlate Positively with Oncogenic Signatures in *Cancer*

**Figure 4:**
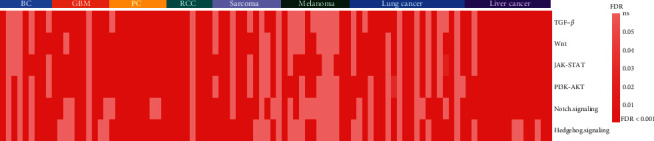
Correlations of ATAXIC scores with oncogenic pathways in cancer. Heatmap showing the FDR of the Spearman correlation between ATAXIC scores and the enrichment scores of the TGF-*β*, Wnt, JAK-STAT, PI3K-Akt, Notch, and Hedgehog signaling pathways in single cells from individual patients of eight cancer types.

We analyzed correlations of ATAXIC scores with several oncogenic pathways in single cancer cells, including TGF-*β*, Wnt, JAK-STAT, PI3K-Akt, Notch, and Hedgehog signaling pathways. Overall, ATAXIC scores likely correlated positively with the enrichment scores of these pathways in single cancer cells. For example, ATAXIC scores had a significant positive correlation with the enrichment scores of TGF-*β* in 6 breast cancers, 9 gliomas, 10 prostate cancers, 7 RCCs, 6 sarcomas, 5 melanomas, 13 lung cancers, and 14 liver cancers (Spearman correlation, FDR < 0.05) (Figure 4 and Supplementary Figure S2A), compared to the significant negative correlation between them in 1 sarcoma and 2 lung cancers. ATAXIC scores significantly and positively correlated with the enrichment scores of Wnt in 7 breast cancers, 8 gliomas, 10 prostate cancers, 7 RCCs, 7 sarcomas, 5 melanomas, 11 lung cancers, and 15 liver cancers (Figure 4 and Supplementary Figure S2B), compared to the significant negative correlation between them in 1 melanoma and 3 lung cancers. In 8 breast cancers, 9 gliomas, 10 prostate cancers, 7 RCCs, 9 sarcomas, 10 melanomas, 11 lung cancers, and 15 liver cancers, there were a significant positive correlation between ATAXIC and JAK-STAT scores (Figure 4 and Supplementary Figure S2C), compared to the significant negative correlation between them in 2 lung cancers. Similar results were observed in the correlations between ATAXIC scores and the enrichment scores of PI3K-Akt, Notch, and Hedgehog (Figure 4 and Supplementary Figure S2D-2F) signaling pathways.

### 3.4. ATAXIC Scores Likely Correlate Positively with DNA Damage Repair Signatures in *Cancer*

**Figure 5:**
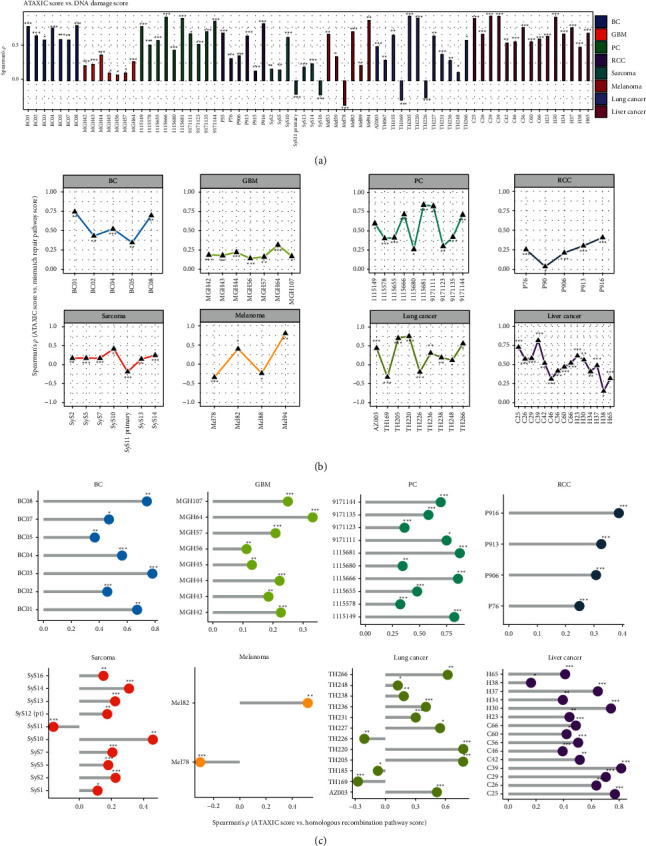
Correlations of ATAXIC scores with DNA damage repair signatures in cancer. Spearman correlations between ATAXIC scores and the enrichment scores of the DNA damage signature (a), the mismatch repair pathway (b), and the homologous recombination pathway (c) in single cells from individual patients of eight cancer types. The Spearman correlation coefficients and adjusted *P* values (FDR) are shown.

We analyzed correlations of ATAXIC scores with DNA damage repair signatures in single cancer cells. First, we found that ATAXIC scores had a significant positive correlation with the scores of DNA damage in 7 breast cancers, 7 gliomas, 10 prostate cancers, 6 RCCs, 5 sarcomas, 5 melanomas, 10 lung cancers, and 15 liver cancers (Spearman correlation, FDR < 0.05) (Figure 5(a)), compared to the significant negative correlation between them in 2 sarcomas, 1 melanoma, and 2 lung cancers. Second, ATAXIC scores were significantly and positively correlated with the enrichment scores of the mismatch repair pathway in 5 breast cancers, 7 gliomas, 10 prostate cancers, 5 RCCs, 6 sarcomas, 2 melanomas, 7 lung cancers, and 15 liver cancers (Figure 5(b)), compared to the significant negative correlation between them in 1 sarcoma, 2 melanomas, and 2 lung cancers. Third, ATAXIC scores had a significant positive correlation with the enrichment scores of the homologous recombination pathway in 7 breast cancers, 8 gliomas, 10 prostate cancers, 4 RCCs, 9 sarcomas, 1 melanoma, 9 lung cancers, and 15 liver cancers (Figure 5(c)), compared to the significant negative correlation between them in 1 sarcoma, 1 melanoma, and 3 lung cancers. The mismatch repair and homologous recombination are two key pathways involved in DNA-damage repair processing in cancer [25]. Thus, our results suggest that ATAXIC scores likely correlate positively with DNA damage repair signatures in cancer.

### 3.5. ATAXIC Scores Likely Correlate Negatively with Clinical Outcomes in *Cancer*

**Figure 6:**
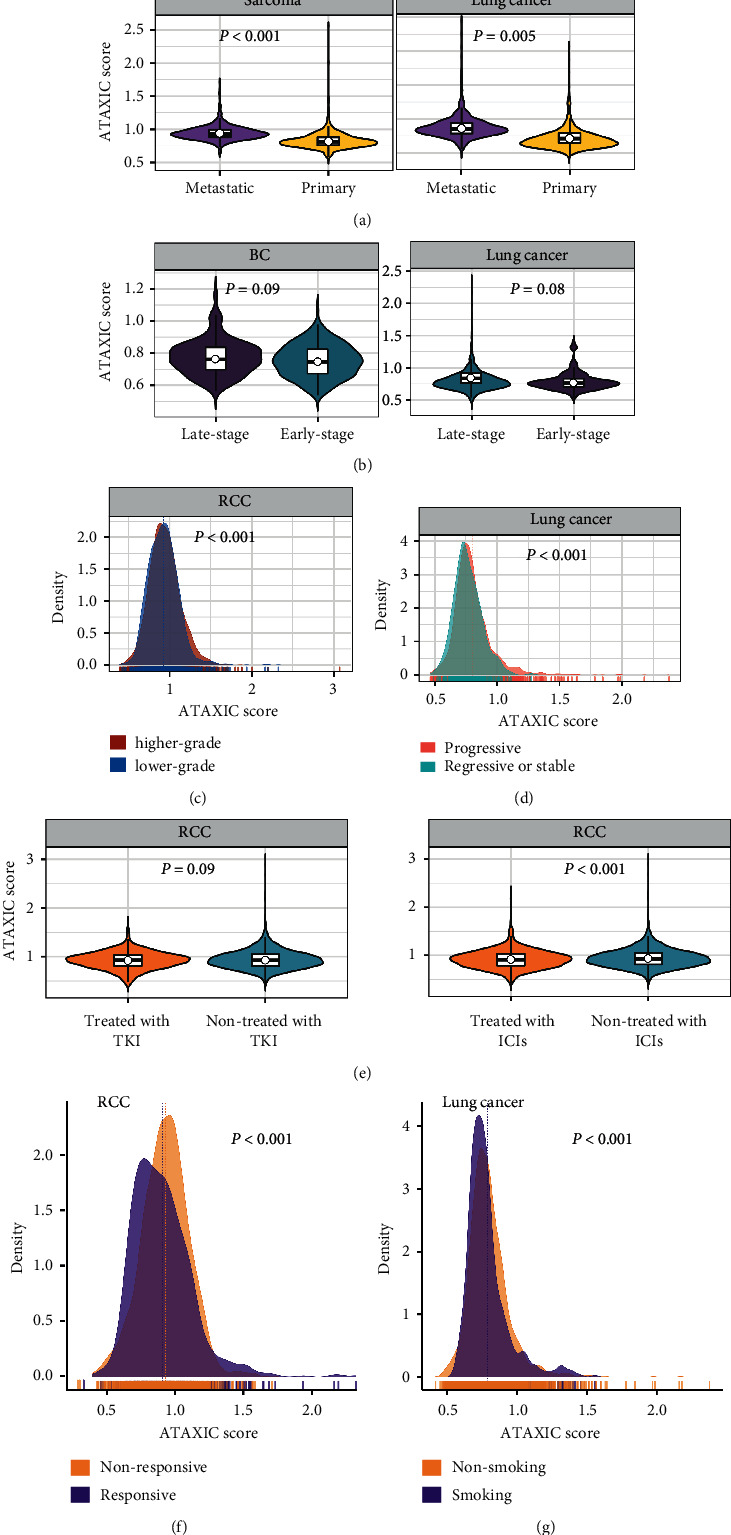
Correlations of ATAXIC scores with clinical characteristics. ATAXIC scores are higher in metastatic versus primary tumors (a), in late-stage versus early-stage tumors (b), in higher-grade (G4) versus lower-grade (G3) tumors (c), and in progressive versus regressive or stable tumors (d). (e) ATAXIC scores are lower in the tumors treated with VEGF inhibitors than those not treated with them and lower in the tumors treated with immune checkpoint inhibitors (ICIs) than those not treated with them in RCC. (f) ATAXIC scores are lower in the tumors responsive to ICIs versus the tumors not responsive to ICIs in RCC. (g) ATAXIC scores are lower in smoker than in nonsmoker lung cancers. The one-tailed Mann–Whitney *U* test *P* values are shown.

We found that ATAXIC scores were significantly higher in metastatic than in primary tumor in sarcoma and lung cancer (one-tailedMann-Whitney*U* test, *P* < 0.01) (Figure 6(a)). This is consistent with the positive association between ATAXIC scores and metastasis signature scores in cancer. In breast cancer and lung cancer, ATAXIC scores tended to be higher in late-stage than in early-stage tumors (*P* < 0.1) (Figure 6(b)). In RCC, ATAXIC scores were significantly higher in higher-grade (G4) than in lower-grade (G3) tumors (*P* < 0.001) (Figure 6(c)). In lung cancer and RCC, ATAXIC scores displayed marked correlations with treatment outcomes. For example, ATAXIC scores were significantly higher in progressive than in regressive or stable lung cancers after treatment (*P* < 0.001) (Figure 6(d)). ATAXIC scores were lower in the tumors treated with VEGF inhibitors than those not treated with them in RCC (*P* < 0.09) (Figure 6(e)). In addition, ATAXIC scores were lower in the tumors treated with immune checkpoint inhibitors (ICIs) than those not treated with them in RCC (*P* < 0.001) (Figure 6(e)). Within the RCC tumors treated with ICIs, ATAXIC scores were lower in the tumors responsive to ICIs versus the tumors not responsive to ICIs (*P* < 0.001) (Figure 6(f)). Overall, these results suggest that the ATAXIC TP heterogeneity is associated with unfavorable clinical outcomes in cancer. Interestingly, ATAXIC scores were significantly lower in smoker than in nonsmoker lung cancers (*P* < 0.001) (Figure 6(g)).

### 3.6. ATAXIC Scores across and within Individual *Cancer* Types

**Figure 7:**
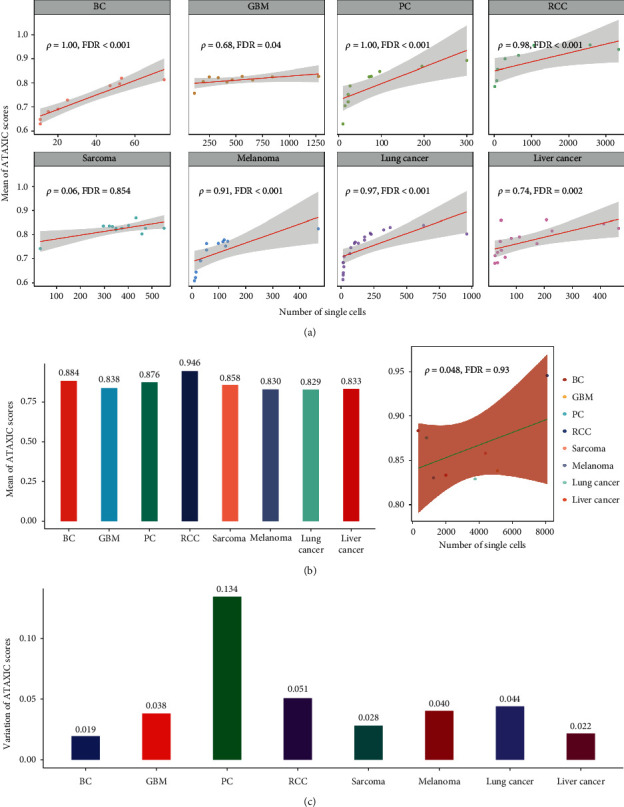
Comparisons of ATAXIC scores across and within individual cancer types. (a) Spearman correlations between ATAXIC scores and the numbers of single cells in individual cancer types. (b) The mean ATAXIC scores of single cells in each of the eight cancer types and the Spearman correlation between the scores and the numbers of single cells in individual cancer types across the eight cancer types. (c) Variations of ATAXIC scores in individual cancer types. The Spearman correlation coefficients (*ρ*) and adjusted *P* values (FDR) are shown in a and b.

We found that the mean ATAXIC score of single cells in a cancer was significantly and positively correlated with its number of single cells in all the individual cancer types except sarcoma (Spearman correlation, FDR < 0.05; *ρ* > 0.68) (Figure 7(a)). It suggests that the average TP heterogeneity level likely increases with the growth of the scale of single cells in individual cancer types. In addition, we calculated the ATAXIC scores of single cells based on the gene expression profiles in all single cells of each cancer type. The mean ATAXIC score of single cells in lung cancer, melanoma, liver cancer, glioma, sarcoma, prostate cancer, breast cancer, and RCC was 0.829, 0.830, 0.833, 0.838, 0.858, 0.876, 0.884, and 0.946, respectively, while it showed no a significant correlation with the number of single cells in each cancer type (FDR = 0.93; *ρ* = 0.048) (Figure 7(b)). It suggests that the significant positive association between the average TP heterogeneity level and the number of single cells within individual cancer types is not established across different cancer types. This disparity may refer to the intertumor heterogeneity among different cancer types. It should be noted that melanoma and lung cancer are two cancer types responding best to ICIs [26], which showed the lowest TP heterogeneity among the eight cancer types. It supports that tumor heterogeneity may hold back antitumor immune response. The variation of ATAXIC scores of single cells varied among these cancer types, with the largest value of 0.134 in prostate cancer and the smallest value of 0.019 in breast cancer (Figure 7(c)). Interestingly, breast cancer had a relatively large mean value but the smallest variation of ATAXIC scores. In contrast, lung cancer had the smallest mean value but a relatively large variation of ATAXIC scores. Again, these results indicate the intertumor heterogeneity among different cancer types.

### 3.7. ATAXIC Scores Likely Correlate Negatively with Drug Sensitivity in *Cancer*

We analyzed correlations between ATAXIC scores and drug sensitivity of 578 cancer cell lines to 4686 compounds using the PRISM Repurposing Primary Screen data from the Dependency Map (DepMap) (https://depmap.org/portal/download/). The viability value of a cancer cell line treated with a compound represents the drug sensitivity of the cancer cell line to the compound. The lower the viability value, the more sensitive the cell line is to the compound. Among the 4686 compounds, 728 showed a significant positive correlation between the viability values and ATAXIC scores in the 578 cancer cell lines, compared to 82 showing a significant negative correlation between them (Supplementary Table S3). These results indicate that ATAXIC scores likely have a significant negative association with drug sensitivity in cancer, supporting that tumor heterogeneity promotes cancer therapeutic resistance [21].

## 4. Discussion

For the first time, we developed an algorithm to evaluate the TP heterogeneity in single cancer cells. It should be noted that this is the first algorithm to measure the heterogeneity of gene expression perturbations in single cancer cells. We revealed that the TP heterogeneity was associated with proliferation and differentiation signatures, various oncogenic signaling, DNA damage repair, treatment resistance, and unfavorable phenotypes and outcomes in cancer. These characteristics in single tumor cells are in line with those displayed in the bulk tumors [27]. Furthermore, we demonstrated that the TP heterogeneity of single cells varied among different cancer types, with lung cancer and melanoma having the lowest average TP heterogeneity level while RCC having the highest average TP heterogeneity level. The low TP heterogeneity in lung cancer and melanoma could bestow relatively higher response rates to ICIs on both cancer types. Interestingly, among the eight cancer types, two most common hormone-associated cancer types (prostate cancer for males and breast cancer for females) displayed significantly different variations of the single cell TP heterogeneity level, which had the large and smallest variations, respectively. Overall, the average TP heterogeneity level has a significant positive correlation with the number of single cells in individual cancer types. It is justified since more tumor cells furnish higher intratumor heterogeneity. However, the average TP heterogeneity level shows no significant positive correlation with the number of single cells across different cancer types. The intertumor heterogeneity among different cancer types could explain for this disparity.

DNA damage repair deficiency often causes genomic instability to drive intratumor heterogeneity [28, 29]. We observed the significant correlations of the single cell TP heterogeneity with DNA damage and its repair signaling pathways, such as mismatch repair and homologous recombination. It confirms the marked associations among DNA damage repair, genomic instability, and tumor heterogeneity at the single cell level. That is, DNA damage repair deficiency as well as genomic instability could be the major source of the TP heterogeneity in single cancer cells.

This study has several limitations. First, because there are relatively small numbers of cancer patient samples in most single scRNA-seq datasets, the analysis of correlations between the TP heterogeneity level and clinical parameters was restricted. Second, due to lack of the data on genomic alterations (such as somatic mutations and copy number alterations) in single cells, the analysis of correlations between the TP heterogeneity level and genomic alterations was missing. Finally, this study investigated only eight cancer types, although the analysis of a wider variety of cancers would strengthen the validity of conclusions.

## 5. Conclusions

ATAXIC is an algorithm to evaluate TP heterogeneity in single cancer cells. The TP heterogeneity by ATAXIC has significant associations with increased cell proliferation, oncogenic signaling, DNA damage repair, treatment resistance, and unfavorable outcomes in cancer. Thus, ATAXIC is a useful algorithm to quantify the TP heterogeneity in single cancer cells and provide new insights into cancer biology as well as potentially valuable markers for cancer diagnosis and treatment.

## Data Availability

The authors declare that all data supporting the findings of this study are available within the paper and its supplementary information files. The ATAXIC scores of single cells of eight cancer types are available at GitHub (https://github.com/XS-Wang-Lab/ATAXIC/).

## Supplementary Materials

Supplementary Materials Table S1: The numbers of patients and their cancer single cells in each cancer type. Table S2: The marker or pathway gene sets of the signatures and pathways analyzed in this study. Table S3: Significant correlations between the viability values and ATAXIC scores in the 578 cancer cell lines for 728 compounds. The Spearman correlation coefficients, *P* values, and adjusted *P* values (FDR) are shown. Figure S1 Spearman correlations between ATAXIC scores and the enrichment scores of the invasion signature in single cells from breast cancer (BC), glioma (GBM), prostate cancer (PC), renal cell carcinoma (RCC), sarcoma, melanoma, and lung cancer. The Spearman correlation coefficients and adjusted *P* values (FDR) are shown. Figure S2: Correlations of ATAXIC scores with oncogenic pathways in cancer. Spearman correlations between ATAXIC scores and the enrichment scores of the TGF-*β* (A), Wnt (B), JAK-STAT (C), PI3K-Akt (D), Notch (E), and Hedgehog signaling pathways (F) in single cells from individual patients of eight cancer types. The Spearman correlation coefficients and adjusted *P* values (FDR) are shown.
