# Supplementary material for: ATAXIC: An Algorithm to Quantify Transcriptomic Perturbation Heterogeneity in Single Cancer Cells
Source: J Oncol. 2022 Aug 31;2022:4106736. doi: 10.1155/2022/4106736 (PMC9452944; doi:10.1155/2022/4106736)
Supplement: Supplementary Materials — Table S1: The numbers of patients and their cancer single cells in each cancer type. Table S2: The marker or pathway gene sets of the signatures and pathways analyzed in this study. Table S3: Significant correlations between the viability values and ATAXIC scores in the 578 cancer cell lines for 728 compounds. The Spearman correlation coefficients, P values, and adjusted P values (FDR) are shown. Figure S1 Spearman correlations between ATAXIC scores and the enrichment scores of the invasion signature in single cells from breast cancer (BC), glioma (GBM), prostate cancer (PC), renal cell carcinoma (RCC), sarcoma, melanoma, and lung cancer. The Spearman correlation coefficients and adjusted P values (FDR) are shown. Figure S2: Correlations of ATAXIC scores with oncogenic pathways in cancer. Spearman correlations between ATAXIC scores and the enrichment scores of the TGF-β (A), Wnt (B), JAK-STAT (C), PI3K-Akt (D), Notch (E), and Hedgehog signaling pathways (F) in single cells from individual patients of eight cancer types. The Spearman correlation coefficients and adjusted P values (FDR) are shown. [file 4106736.f1.zip › Table S1.pdf]

Table S1.The The numbers of patients and their cancer single cells in each cancer type.

Cancer type	Number of cancer cell patients	Patient ID of cancer cells	Number of cancer cells	Sequencing technique	Reference
Breast cancer (BC)	9	BC01(ER+)	20	SMARTer	[1]
		BC02 (ER+)	53		
		BC03 (ER+/HER+)	25		
		BC04 (HER2+)	47		
		BC05 (HER2+)	75		
		BC07 (TNBC)	52		
		BC08 (TNBC)	15		
		BC10 (TNBC)	11		
		BC11 (TNBC)	11		
Glioma (GBM)	10	MGH42	466	Smart-seq2	[2]
		MGH43	249		
		MGH44	557		
		MGH45	416		
		MGH56	842		
		MGH57	334		
		MGH61	1273		
		MGH64	656		
		MGH103	112		
		MGH107	192		
Prostate cancer (PC)	10	1115149	15	Smart-seq2	[3]
		1115578	300		
		1115655	195		
		1115666	22		
		1115680	71		
		1115681	26		
		9171111	10		
		9171123	96		
		9171135	76		
		9171144	22		
Renal cell carcinoma (RCC)	8	P55	93	10× platform	[4]
		P76	2573		
		P90	3334		
		P906	1065		
		P912	16		
		P913	284		

		P915	649		
		P916	70		
Sarcoma	12	SyS1	349	Smart-seq2	[5]
		SyS2	321		
		SyS5	433		
		SyS7	402		
		SyS10	33		
		SyS11 (metastatic)	473		
		SyS11 （primary）	458		
		SyS12 (pt)	334		
		SyS12	550		
		SyS13	349		
		SyS14	373		
		SyS16	296		
Melanoma	12	Mel53	16	Smart-seq2	[6]
		Mel59	54		
		Mel71	54		
		Mel78	120		
		Mel79	468		
		Mel80	125		
		Mel81	133		
		Mel82	32		
		Mel84	14		
		Mel88	115		
		Mel89	98		
		Mel94	10		
Lung cancer	20	AZ003	73	Smart-seq2	[7]
		TH067	100		
		TH103	66		
		TH155	17		
		TH169	179		
		TH171	629		
		TH179	131		
		TH185	958		
		TH205	24		
		TH210	15		
		TH218	18		
		TH220	178		
		TH225	22		
		TH226	222		
		TH227	17		

		TH231	325		
		TH236	107		
		TH238	229		
		TH248	375		
		TH266	18		
Liver cancer	15	C25	111	10× platform	[8]
		C26	174		
		C29	34		
		C39	24		
		C42	34		
		C46	464		
		C56	82		
		C60	47		
		C66	208		
		H23	46		
		H30	25		
		H34	43		
		H37	60		
		H38	228		
		H65	412		

Reference

[1] Chung, W., et al. Single-cell RNA-seq enables comprehensive tumour and immune cell profiling in primary breast cancer. Nat Commun 8, 15081 (2017).

[2] Venteicher, A.S., et al. Decoupling genetics, lineages, and microenvironment in IDH-mutant gliomas by single-cell RNA-seq. Science 355(2017).

[3] He, M.X., et al. Transcriptional mediators of treatment resistance in lethal prostate cancer. Nat Med 27, 426-433 (2021).

[4] Bi, K., et al. Tumor and immune reprogramming during immunotherapy in advanced renal cell carcinoma. Cancer Cell 39, 649-661 e645 (2021).

[5] https://www.ncbi.nlm.nih.gov/geo/query/acc.cgi?acc=GSE131309

[6] Tirosh, I., et al. Dissecting the multicellular ecosystem of metastatic melanoma by single-cell RNA-seq. Science 352, 189-196 (2016).

[7] Maynard, A., et al. Therapy-Induced Evolution of Human Lung Cancer Revealed by Single-Cell RNA Sequencing. Cell 182, 1232-1251 e1222 (2020).

[8] Ma, L., et al. Tumor Cell Biodiversity Drives Microenvironmental Reprogramming in Liver Cancer. Cancer Cell 36, 418-430 e416 (2019).
