# Supplementary material for: ATAXIC: An Algorithm to Quantify Transcriptomic Perturbation Heterogeneity in Single Cancer Cells
Source: J Oncol. 2022 Aug 31;2022:4106736. doi: 10.1155/2022/4106736 (PMC9452944; doi:10.1155/2022/4106736)
Supplement: Supplementary Materials — Table S1: The numbers of patients and their cancer single cells in each cancer type. Table S2: The marker or pathway gene sets of the signatures and pathways analyzed in this study. Table S3: Significant correlations between the viability values and ATAXIC scores in the 578 cancer cell lines for 728 compounds. The Spearman correlation coefficients, P values, and adjusted P values (FDR) are shown. Figure S1 Spearman correlations between ATAXIC scores and the enrichment scores of the invasion signature in single cells from breast cancer (BC), glioma (GBM), prostate cancer (PC), renal cell carcinoma (RCC), sarcoma, melanoma, and lung cancer. The Spearman correlation coefficients and adjusted P values (FDR) are shown. Figure S2: Correlations of ATAXIC scores with oncogenic pathways in cancer. Spearman correlations between ATAXIC scores and the enrichment scores of the TGF-β (A), Wnt (B), JAK-STAT (C), PI3K-Akt (D), Notch (E), and Hedgehog signaling pathways (F) in single cells from individual patients of eight cancer types. The Spearman correlation coefficients and adjusted P values (FDR) are shown. [file 4106736.f1.zip › Table S2.pdf]

Table S2.The marker or pathway gene sets of the signatures and pathways analyzed in this study.

Proliferation	*ADK, BAX, CCND1, BMPR1A, BUB1B, CCNA2, CCNB1, CD40, CDK1, CDC20, CYBA, HMGB2, IGFBP2, MKI67, PRKD1, TEK, TK1, TOP2A, DLGAP5, MELK, GINS1, KIF14, RACGAP1, ASPM, CD24, CX3CL1, IGF1, CD74, TP63, FGFR1, DCT, PTPRC, LHX5, ESR1, ABL1, XBP1, PDGFB, HIF1A, CD40LG, FGF9, FZD3, RIPK2, NUMBL, EBI3, WNT2, IL2, IL23A, VEGFA, SEMA5A, IL4, CD86, STAT1, ID2, DOCK7, SLAMF1, TNFSF4, WNT1, TNFSF9, MED1, SMO, TLR4, MYC, LEF1, ASCL1, FGF7, OSR1, IL18, WNT3A, WNT7A, DISC1, BMP10, SYK, IL13, BCL2, ID4, STAT5B, CD34, CTC1, TBX1, IRS2, TNFRSF4, HMGB1, ZNF335, NRARP, BMPR2, AGER, MIF, CCL5*
Cell cycle	*CCND1, CCND2, CCND3, CDK4, CDK6, RB1, RBL1, RBL2, ABL1, HDAC1, HDAC2, E2F1, E2F2, E2F3, E2F4, E2F5, TFDP1, TFDP2, GSK3B, TGFB1, TGFB2, TGFB3, SMAD2, SMAD3, SMAD4, MYC, ZBTB17, CDKN2A, CDKN2B, CDKN2C, CDKN2D, CDKN1B, CDKN1C, CDKN1A, CCNE1, CCNE2, CDK2, SKP1, CUL1, RBX1, SKP2, CCNA2, CCNA1, CDC6, CDC45, CDC7, DBF4, CDK1, CCNB1, CCNB2, CCNB3, CDC25B, CDC25C, YWHAZ, YWHAB, YWHAQ, YWHAE, YWHAH, YWHAG, PLK1, WEE1, WEE2, PKMYT1, CCNH, CDK7, ANAPC1, ANAPC2, CDC27, ANAPC4, ANAPC5, CDC16, ANAPC7, CDC23, ANAPC10, ANAPC11, CDC26, ANAPC13, CDC20, PTTG1, PTTG2, ESPL1, SMC1A, SMC1B, SMC3, STAG2, STAG1, RAD21, TTK, BUB1, BUB3, BUB1B, MAD1L1, MAD2L1, MAD2L2, FZR1, CDC14B, CDC14A, ATR, ATM, TP53, CHEK1, CHEK2, CREBBP, EP300, PRKDC, MDM2, GADD45A, GADD45B, GADD45G, PCNA, SFN, CDC25A, ORC1, ORC2, ORC3, ORC4, ORC5, ORC6, MCM2, MCM3, MCM4, MCM5, MCM6, MCM7*
Differentiation	*ABL1, ACVR1, ADA, AGT, AKT1, ANXA1, ATOH1, ATP2B2, ATP7A, AXL, BCL2, BCL3, PRDM1, BMP2, BMP6, BRAF, CASP8, CAV3, RUNX2, RUNX1, CCNB1, CD86, CDK1, CDC42, CDH2, CEBPA, CEBPB, CREB1, MAPK14, CSF1, CSF1R, CSF2, DRD1, EDN3, EDNRB, EPHA2, EMX1, EP300, ERBB4, ERCC2, FGF8, FGFR1, FGFR2, FOXC2, FLT3LG, MTOR, GATA1, GATA4, GATA6, GDNF, GNAS, HDAC2, NCKAP1L, NRG1, HHEX, HIF1A, HMGB1, FOXA1, HOXA5, IGF1, IL2, IL4R, IL11, IL12B, IL15, ILK, INSM1, IRF4, JAG2, JUN, JUNB, KIT, LAMB2, LEP, LGALS1, LGALS9, LHX1, LIF, LMNA, LYN, SMAD4, MEF2A, MEF2C, KITLG, MMP14, MYF5, MYF6, MYH9, MYO7A, MYOD1, MYOG, NFATC2, NFIB, NTRK1, NTRK2, PAFAH1B1, PAX2, PF4, PITX2, PLS3, POU4F3, PPARG, MAPK3, MAP2K1, PROX1, PSEN1, PTGER4, PTN, PTPN11, PTPRZ1, RAC1, RAG1, RB1, RELB, RXRA, CCL19, SHOX2, SNAI1, SOD1, SOX4, SOX10, SOX11, SP3, SPI1, SRF, STAT5B, STK11, SYK, TAL1, TGFB2, TGFBR2, THRA, NKX2-1, TRAF6, TSC1, VEGFA, WNT7A, WNT8A, WNT11, WT1, ZAP70, EOMES, SEMA7A, TNFSF11, TP63, TNFSF9, FADD, NRP1, PROM1, SLC9A3R1, ATG5, SLC4A7, MAML1, ZEB2, TSPAN2, SEC24B, BATF, ZBTB1, KDM1A, KDM6B, HEY2, SCRIB, TENM4, BLOC1S6, FOXP1, BMP10, IL20, CDON, WDPCP, LEF1, IL23A, CLIC5, DLL4, TMEM100, MCOLN3, SOX6, MESP1, CYP26B1, FAM20C, OVOL2, SAV1, SEMA4A, BCL11B, NKAP, PDZD7, WNT5B, DCSTAMP, GDPD5, SLITRK6, MYLK3, TMC1, OCSTAMP, IL31RA, IL23R, GDF7, NKX2-3, STRC, ARX, GSX2, LHFPL5, ATP11C*
Invasion	*AEBP1, AKR1B1, AMD1, SLC25A5, ATP5PB, BAG1, BGN, C1QB, CALD1, CAPG, CCNE1, CDH11, CKS1B, CKS2, COL1A1, COL1A2, COL3A1, COL5A1, COL5A2, COL6A2, COL6A3, COL10A1, COL11A1, COMP, CSE1L, VCAN, CTSK, DAB2, DDX5, EDNRA, FAP, FBN1, FN1, GNAS, H2AFZ, HMGB2, HNRNPU, HSD17B4, CYR61, INHBA, LAMB1, LAMC1, LGALS1, LOX, LOXL2, LUM, MMP2, MMP11, HNRNPM, NDUFB7, YBX1, PDGFRB, PLAU, PRRX1, PNN, PPIC, PROS1, PSMA2, PSMB4, RGS4, SNAI2, SPOCK1, TGFBI, THBS2, THY1, TNFAIP6, UBE2V2, ADAM12, MFAP5, ITGBL1, TP53I3, NUAK1, HNRNPDL, TXNDC9, LRRC17, IFI30, POSTN, CBX1, NID2, RRAS2, RALY, SEPHS2, HEY1, MXRA5, OLFML2B, TMEM158, WWTR1, GREM1, NOX4, CLEC4A, COPZ2, ASPN, CEMIP, CRISPLD2, TUBB6, LRRC15, TUBB*
Metastasis	*ACKR3, AFP, AGR2, AKT1, ALDH1A1, ANGPTL4, ANXA1, ANXA2, AQP5, ATF3, AURKA, AXL, B2M, BMI1, BMP2, BRAF, BSG, CA9, CCDC88A, CCND1, CCR7, CD24, CD274, CD44, CDCP1, CDH17, CDH2, CEACAM1, CEACAM5, CLIC1, CRP, CTGF, CTNND1, CTSB, CTSK, CTSL, CTTN, CXCL12, CXCL2, CXCL8, CXCR2, CYR61, DDR2, EGF, EGFR, EGR1, EPAS1, EPCAM, ERBB2, ERBB3, ESR1, ESR2, EZH2, EZR, F2RL1, FGFR1, FLOT2, FLT1, FLT4, FN1, FOXC2, FOXM1, GLI1, HGF, HIF1A, HMGA1, HMGA2, HPSE, ID1, IDO1, IGF1R, IGF2BP3, IL6, ITGA3, ITGA5, ITGA6, ITGAV, ITGB1, ITGB3, JAG1, KDR, KRAS, KRT19, L1CAM, LAMC2, LASP1, LGALS3, LOX, LOXL2, MACC1, MCAM, MDM2, MET, MIF, MKI67, MME, MMP1, MMP11, MMP13, MMP14, MMP2, MMP3, MMP7, MMP9, MSN, MST1R, MTA1, MTDH, MUC1, MYC, NEDD9, NOTCH1, NTRK2, PCNA, PDGFRA, PDPN, PIK3CA, PKM, PLAU, PLAUR, POSTN, PRKCI, PROM1, PSCA, PTGS2, PTHLH, PTK2, PTP4A3, PTTG1, PXN, RAB25, RAC1, RELA, RHOC, ROCK1, S100A4, SATB1, SDCBP, SELE, SELP, SLC2A1, SNAI1, SNAI2, SNCG, SOX4, SPARC, SPP1, SRC, STAT3, STMN1, TERT, TGFBI, TGM2, TIMP1, TMPRSS4, TNFSF11, TYMP, VCAN, VEGFA, VEGFC, VEGFD, VIM, WASF3, YAP1, ZEB1, ZEB2*
Stemness	*AFMID, AFP, ANPEP, APC, AQP1, ASCL1, ASCL2, AXIN2, AZGP1, BAZ2B, BMI1, BOC, C6orf62, CA2, CAMK2N1, CCL5, CCND2, CD200, CD24, CD33, CD38, CD44, CDCA7, CDK6, CEBPA, CFTR, CHD7, CORO1C, CXCL2, DBX1, DBX2, DNMT3A, DPP4, EBF1, EEF1A1, EGFR, EIF4B, EMX1, EMX2, ENG, EPHB2, ETS2, ETV1, EVI2A, EZH2, FABP7, FAM84A, FBLIM1, FBXO27, FERMT1, FOXA2, FOXA3, FOXG1, GATA1, GATA2, GATA3, GATA4, GFAP, GFI1, GLI1, GPC3, GPM6A, GPSM2, GPX2, H19, H3F3B, HACD3, HAPLN1, HEPN1, HES1, HNRNPH1, HNRNPL, HOPX, ICAM1, IDH1, IKZF1, IRX3, ITGAM, KDR, KIT, KLK10, KRT14, LATS2, LGR5, LOH12CR2, LRIG1, LY6D, LYZ, MALAT1, MBOAT1, MESP1, MESP2, METTL3, MLLT10, MME, MYB, MYC, NANOG, NCAM1, NEK5, NELL2, NFE2, NFIA, NFIB, NKX2-5, NODAL, NT5E, OLFM4, OPHN1, ORC6, PABPC1, PAX6, POU5F1, PROM1, PTK7, PTMA, PTPRC, PTPRG, PTPRO, PTPRS, PTPRZ1, QPCTL, RAB42, RAMP2-AS1, RBM6, RGMB, RNF43, RUNX1, SET, SLC12A2, SMAD2, SMOC2, SOX1, SOX11, SOX17, SOX2, SOX3, SOX4, SOX9, SPDYE1, SPDYE5, SPHKAP, SRGAP2C, STMN1, STMN2, TAL1, TATDN3, TCF12, TCF4, TDGF1, TFDP2, TFRC, THY1, TNFAIP8L1, TOX3, TRA2A, TSPAN6, UGT8, VEGFA, ZBTB8A, ZNF793, ABCG2, ALDH1A1, ALDH1A3, DNER, MET*
TGF.β.signaling	*CHRD, NOG, NBL1, MICOS10-NBL1, GREM1, GREM2, THBS1, DCN, FMOD, LEFTY1, LEFTY2, FST, BMP2, BMP4, BMP6, INHBB, BMP5, BMP7, BMP8B, BMP8A, GDF5, GDF6, GDF7, AMH, THSD4, FBN1, LTBP1, TGFB1, TGFB2, TGFB3, INHBA, INHBC, INHBE, NODAL, NEO1, HJV, BMPR1A, BMPR1B, ACVR1, BMPR2, ACVR2A, RGMA, RGMB, AMHR2, TGFBR1, TGFBR2, ACVR1B, ACVR2B, ACVR1C, BAMBI, SMAD1, SMAD5, SMAD9, SMAD2, SMAD3, SMAD4, SMAD6, SMAD7, SMURF1, SMURF2, ZFYVE9, ZFYVE16, HAMP, ID1, ID2, ID3, ID4, RBL1, E2F4, E2F5, TFDP1, CREBBP, EP300, SP1, TGIF1, TGIF2, MYC, CDKN2B, PITX2, RBX1, CUL1, SKP1, MAPK1, MAPK3, IFNG, TNF, RHOA, ROCK1, PPP2R1B, PPP2R1A, PPP2CA, PPP2CB, RPS6KB1, RPS6KB2*
Wnt signaling	*PORCN, WNT1, WNT2, WNT2B, WNT3, WNT3A, WNT4, WNT5A, WNT5B, WNT6, WNT7A, WNT7B, WNT8A, WNT8B, WNT9A, WNT9B, WNT10B, WNT10A, WNT11, WNT16, CER1, NOTUM, WIF1, SERPINF1, SOST, DKK1, DKK2, DKK4, SFRP1, SFRP2, SFRP4, SFRP5, RSPO1, RSPO2, RSPO3, RSPO4, LGR4, LGR5, LGR6, RNF43, ZNRF3, FZD1, FZD7, FZD2, FZD3, FZD4, FZD5, FZD8, FZD6, FZD10, FZD9, LRP5, LRP6, BAMBI, CSNK1E, TPTEP2-CSNK1E, DVL3, DVL2, DVL1, FRAT1, FRAT2, CSNK2A1, CSNK2A2, CSNK2A3, CSNK2B, NKD1, NKD2, CXXC4, SENP2, GSK3B, CTNNB1, AXIN1, AXIN2, APC, APC2, CSNK1A1L, CSNK1A1, TCF7, TCF7L1, TCF7L2, LEF1, CTNNBIP1, CBY1, CHD8, SOX17, CTBP1, CTBP2, CTNND2, CREBBP, EP300, RUVBL1, SMAD4, SMAD3, MAP3K7, NLK, MYC, JUN, FOSL1, CCND1, CCND2, CCND3, CCN4, PPARD, MMP7, PSEN1, PRKACA, PRKACB, PRKACG, TP53, SIAH1, CACYBP, SKP1, TBL1X, TBL1Y, TBL1XR1, BTRC, FBXW11, CUL1, RBX1, GPC4, ROR1, ROR2, RYK, VANGL2, VANGL1, PRICKLE1, PRICKLE2, PRICKLE4, PRICKLE3, INVS, DAAM1, DAAM2, RHOA, ROCK2, RAC1, RAC2, RAC3, MAPK8, MAPK10, MAPK9, PLCB1, PLCB2, PLCB3, PLCB4, CAMK2A, CAMK2D, CAMK2B, CAMK2G, PPP3CA, PPP3CB, PPP3CC, PPP3R1, PPP3R2, PRKCA, PRKCB, PRKCG, NFATC1, NFATC2, NFATC3, NFATC4*

PI3K-Akt signaling	*EGF, TGFA, EREG, AREG, FGF1, FGF2, FGF3, FGF4, FGF17, FGF6, FGF7, FGF8, FGF9, FGF10, FGF16, FGF5, FGF18, FGF20, FGF22, FGF19, FGF21, FGF23, NGF, BDNF, NTF3, NTF4, INS, IGF1, IGF2, PDGFA, PDGFB, PDGFC, PDGFD, CSF1, KITLG, FLT3LG, VEGFA, VEGFB, PGF, VEGFC, VEGFD, HGF, ANGPT1, ANGPT2, ANGPT4, EFNA1, EFNA2, EFNA3, EFNA4, EFNA5, EGFR, ERBB2, ERBB3, ERBB4, FGFR1, FGFR2, FGFR3, FGFR4, NGFR, NTRK1, NTRK2, INSR, IGF1R, PDGFRA, PDGFRB, CSF1R, KIT, FLT3, FLT1, FLT4, KDR, MET, TEK, EPHA2, GRB2, SOS1, SOS2, HRAS, KRAS, NRAS, RAF1, MAP2K1, MAP2K2, MAPK1, MAPK3, IRS1, TLR2, TLR4, RAC1, IGH, SYK, CD19, PIK3AP1, GH1, GH2, CSH1, CSH2, PRL, OSM, IL2, IL3, IL6, IL4, IL7, IFNA1, IFNA2, IFNA4, IFNA5, IFNA6, IFNA7, IFNA8, IFNA10, IFNA13, IFNA14, IFNA16, IFNA17, IFNA21, IFNB1, EPO, CSF3, GHR, PRLR, OSMR, IL2RA, IL2RB, IL2RG, IL3RA, IL6R, IL4R, IL7R, IFNAR1, IFNAR2, EPOR, CSF3R, JAK1, JAK2, JAK3, COL1A1, COL1A2, COL2A1, COL4A2, COL4A4, COL4A6, COL4A1, COL4A5, COL4A3, COL6A1, COL6A2, COL6A3, COL6A6, COL6A5, COL9A1, COL9A2, COL9A3, LAMA1, LAMA2, LAMA3, LAMA5, LAMA4, LAMB1, LAMB2, LAMB3, LAMB4, LAMC1, LAMC2, LAMC3, CHAD, RELN, THBS1, COMP, THBS2, THBS3, THBS4, FN1, SPP1, VTN, TNC, TNN, TNR, TNXB, VWF, IBSP, ITGA1, ITGA2, ITGA2B, ITGA3, ITGA4, ITGA5, ITGA6, ITGA7, ITGA8, ITGA9, ITGA10, ITGA11, ITGAV, ITGB1, ITGB3, ITGB4, ITGB5, ITGB6, ITGB7, ITGB8, PTK2, PIK3CA, PIK3CD, PIK3CB, PIK3R1, PIK3R2, PIK3R3, F2R, CHRM1, CHRM2, LPAR1, LPAR2, LPAR3, LPAR4, LPAR5, LPAR6, GNB1, GNB2, GNB3, GNB4, GNB5, GNG2, GNG3, GNG4, GNG5, GNG7, GNG8, GNG10, GNG11, GNG12, GNG13, GNGT1, GNGT2, PIK3CG, PIK3R5, PIK3R6, PDPK1, STK11, PRKAA1, PRKAA2, DDIT4, TSC1, TSC2, RHEB, MLST8, MTOR, RPTOR, EIF4EBP1, EIF4E, EIF4E2, EIF4E1B, RPS6KB1, RPS6KB2, EIF4B, RPS6, PRKCA, PKN1, PKN2, PKN3, SGK1, SGK2, SGK3, C8orf44-SGK3, AKT1, AKT2, AKT3, MAGI1, MAGI2, PTEN, THEM4, PPP2CA, PPP2CB, PPP2R1B, PPP2R1A, PPP2R2A, PPP2R2B, PPP2R2C, PPP2R2D, PPP2R3B, PPP2R3C, PPP2R3A, PPP2R5B, PPP2R5C, PPP2R5D, PPP2R5E, PPP2R5A, HSP90AA1, HSP90AB1, HSP90B1, CDC37, CRTC2, PHLPP1, PHLPP2, TCL1A, TCL1B, MTCP1, NOS3, BRCA1, GSK3B, GYS2, GYS1, PCK1, PCK2, G6PC, G6PC2, G6PC3, MYC, CCND1, CDKN1A, CDKN1B, CDK2, CDK4, CDK6, CCND2, CCND3, CCNE1, CCNE2, FOXO3, RBL2, FASLG, BCL2L11, YWHAZ, YWHAB, YWHAQ, YWHAE, YWHAH, YWHAG, BAD, BCL2L1, BCL2, CASP9, CREB1, ATF2, ATF4, CREB3, CREB3L1, CREB3L2, CREB3L3, CREB3L4, CREB5, ATF6B, MCL1, RXRA, NR4A1, IKBKG, CHUK, IKBKB, RELA, NFKB1, MYB, MDM2, TP53*
JAK-STAT signaling	*IL2, IL3, IL4, IL5, IL6, IL7, IL9, IL10, IL11, IL12A, IL12B, IL13, IL15, IL17D, IL19, IL20, IL21, IL22, IL23A, IL24, IFNA1, IFNA2, IFNA4, IFNA5, IFNA6, IFNA7, IFNA8, IFNA10, IFNA13, IFNA14, IFNA16, IFNA17, IFNA21, IFNB1, IFNG, IFNE, IFNK, IFNL1, IFNL2, IFNL3, IFNW1, OSM, LIF, TSLP, CTF1, CSF2, CNTF, CSF3, EPO, GH1, GH2, CSH1, CSH2, LEP, THPO, PRL, EGF, PDGFA, PDGFB, IL2RA, IL2RB, IL2RG, IL3RA, IL4R, IL5RA, IL6R, IL7R, IL9R, IL10RA, IL10RB, IL11RA, IL12RB1, IL12RB2, IL13RA1, IL13RA2, IL15RA, IL20RA, IL20RB, IL21R, IL22RA1, IL22RA2, IL23R, IL27RA, IL6ST, IFNAR1, IFNAR2, IFNGR1, IFNGR2, IFNLR1, OSMR, LIFR, CRLF2, CNTFR, CSF2RA, CSF2RB, CSF3R, EPOR, GHR, LEPR, MPL, PRLR, EGFR, PDGFRA, PDGFRB, JAK1, JAK2, JAK3, TYK2, STAT1, STAT2, STAT3, STAT4, STAT5A, STAT5B, STAT6, CISH, SOCS1, SOCS2, SOCS3, SOCS4, SOCS5, SOCS7, SOCS6, BCL2, MCL1, BCL2L1, PIM1, MYC, CCND1, CCND2, CCND3, CDKN1A, AOX1, GFAP, STAM2, STAM, PTPN2, PTPN6, IRF9, CREBBP, EP300, PIAS1, PIAS2, PIAS3, PIAS4, FHL1, PTPN11, GRB2, SOS1, SOS2, HRAS, RAF1, PIK3CA, PIK3CD, PIK3CB, PIK3R1, PIK3R2, PIK3R3, AKT1, AKT2, AKT3, MTOR*
Notch signaling	*DLL3, DLL1, DLL4, JAG1, JAG2, MFNG, LFNG, RFNG, NOTCH1, NOTCH2, NOTCH3, NOTCH4, RBPJL, RBPJ, HES1, HES5, HEYL, HEY1, HEY2, PTCRA, DVL3, DVL2, DVL1, NUMB, NUMBL, DTX2, DTX3L, DTX1, DTX3, DTX4, ADAM17, PSEN1, PSEN2, PSENEN, NCSTN, APH1A, APH1B, MAML3, MAML2, MAML1, CREBBP, EP300, KAT2B, KAT2A, SNW1, CTBP1, CTBP2, TLE7, TLE1, TLE2, TLE3, TLE4, TLE6, NCOR2, CIR1, HDAC1, HDAC2, ATXN1L, ATXN1*
Hedgehog signaling	*PTCH1, PTCH2, SMO, GPR161, PRKACA, PRKACB, PRKACG, CSNK1A1L, CSNK1A1, CSNK1G2, CSNK1G3, CSNK1G1, CSNK1D, CSNK1E, TPTEP2-CSNK1E, GSK3B, GLI1, GLI2, GLI3, SUFU, KIF7, HHIP, CCND1, CCND2, BCL2, CUL1, BTRC, FBXW11, HHAT, HHATL, SHH, IHH, DHH, DISP1, SCUBE2, BOC, CDON, GAS1, LRP2, MOSMO, MEGF8, MGRN1, SMURF1, SMURF2, GRK2, GRK3, EVC, EVC2, EFCAB7, IQCE, ARRB1, ARRB2, KIF3A, CUL3, SPOP, SPOPL*
DNA damage	*BRCA1, MRE11, FOXN3, HIPK2, SP100, PSME4, RPS6KA6, TP63, GTSE1, PPP2R5C, TP73, CNOT4, DNAJA1, BAX, UIMC1, AURKA, CNOT3, PCBP4, TFAP4, E2F1, RGCC, RBL2, NBN, GML, BABAM1, CASP2, CDK5RAP3, CCND1, FOXM1, CDKN1B, CNOT2, ING4, CNOT6, TFDP2, NEK11, PSMD14, ARID3A, MAD2L2, GADD45A, SYF2, RPA2, PLAGL1, RBBP6, CDK2, CDKN1A, SOX4, CNOT1, PRMT1, BCL2L2, PCNA, RBM38, CCNB1, TAOK3, MDM2, CNOT6L, BRCA2, PML, FANCI, TP53, PMAIP1, CARM1, DTL, RPS27A, CNOT9, CCNA2, PLK2, TNKS1BP1, RPS3, ATM, CHEK1, HMGA2, UBC, CENPJ, XPC, CNOT8, BABAM2, CDC25C, CNOT11, BTG2, ZNF385A, USP1, ABRAXAS1, RNF168, SPIDR, E2F7, CRADD, CDK1, UBB, TRIAP1, RAD9A, KAT5, PLK3, ATR, SFN, HIC1, PIDD1, UBE2N, NPM1, CNOT10, CHEK2, FOXO4, MUC1, BRCC3, BLM, TFDP1, MDM4, CNOT7, E2F4, UBA52, PRKDC*
Mismatch repair	*SSBP1, PMS2, MLH1, MSH6, MSH2, MSH3, MLH3, RFC1, RFC4, RFC2, RFC5, RFC3, PCNA, EXO1, RPA1, RPA2, RPA3, RPA4, POLD1, POLD2, POLD3, POLD4, LIG1*
Homologous recombination	*SSBP1, RAD50, MRE11, NBN, ATM, BRCA1, BARD1, RBBP8, BRIP1, TOPBP1, ABRAXAS1, UIMC1, BABAM1, BABAM2, BRCC3, PALB2, BRCA2, SEM1, SYCP3, RPA1, RPA2, RPA3, RPA4, RAD51, RAD52, RAD51B, RAD51C, RAD51D, XRCC2, XRCC3, RAD54L, RAD54B, POLD1, POLD2, POLD3, POLD4, BLM, TOP3A, TOP3B, MUS81, EME1*
