# Supplementary material for: ATAXIC: An Algorithm to Quantify Transcriptomic Perturbation Heterogeneity in Single Cancer Cells
Source: J Oncol. 2022 Aug 31;2022:4106736. doi: 10.1155/2022/4106736 (PMC9452944; doi:10.1155/2022/4106736)
Supplement: Supplementary Materials — Table S1: The numbers of patients and their cancer single cells in each cancer type. Table S2: The marker or pathway gene sets of the signatures and pathways analyzed in this study. Table S3: Significant correlations between the viability values and ATAXIC scores in the 578 cancer cell lines for 728 compounds. The Spearman correlation coefficients, P values, and adjusted P values (FDR) are shown. Figure S1 Spearman correlations between ATAXIC scores and the enrichment scores of the invasion signature in single cells from breast cancer (BC), glioma (GBM), prostate cancer (PC), renal cell carcinoma (RCC), sarcoma, melanoma, and lung cancer. The Spearman correlation coefficients and adjusted P values (FDR) are shown. Figure S2: Correlations of ATAXIC scores with oncogenic pathways in cancer. Spearman correlations between ATAXIC scores and the enrichment scores of the TGF-β (A), Wnt (B), JAK-STAT (C), PI3K-Akt (D), Notch (E), and Hedgehog signaling pathways (F) in single cells from individual patients of eight cancer types. The Spearman correlation coefficients and adjusted P values (FDR) are shown. [file 4106736.f1.zip › Table S3.pdf]

Table S3. Significant correlations between the viability values and ATAXIC scores in the 578 cancer cell lines for 728 compounds. The Spearman correlation coefficients, P values and adjusted P values (FDR) are shown.

**Drug**	***ρ***	***P* value**	**FDR**
BRD-K07612980-001-05-3::2.59::HTS	0.2961	6.34E-12	2.97E-08
BRD-K18518344-001-15-9::2.5::HTS	0.2777	4.87E-11	1.14E-07
BRD-K60348325-065-09-9::2.5::HTS	0.2695	2.46E-10	1.92E-07
BRD-K76703230-004-03-3::2.5::HTS	0.2693	1.89E-10	2.95E-07
BRD-K69650333-003-14-0::2.5::HTS	0.2692	1.90E-10	2.23E-07
BRD-K37379014-001-01-2::2.5::HTS	0.2687	2.06E-10	1.93E-07
BRD-K58435339-001-03-0::2.5::HTS	0.2660	2.53E-10	1.69E-07
BRD-K29415052-050-04-8::2.5::HTS	0.2643	3.06E-10	1.79E-07
BRD-K18961567-001-01-1::2.5::HTS	0.2601	7.89E-10	4.11E-07
BRD-A66419424-001-02-4::2.5::HTS	0.2586	1.06E-09	4.96E-07
BRD-K41859756-001-06-8::2.5::HTS	0.2560	1.47E-09	5.76E-07
BRD-K71281111-001-04-3::2.5::HTS	0.2557	1.26E-09	5.37E-07
BRD-A55484088-050-02-5::2.5::HTS	0.2538	1.73E-09	5.78E-07
BRD-K71467466-001-02-3::2.5::HTS	0.2532	1.70E-09	6.11E-07
BRD-K86525559-001-07-8::2.52::HTS	0.2512	6.64E-09	1.48E-06
BRD-K51911221-001-01-2::2.5::HTS	0.2511	2.35E-09	7.33E-07
BRD-K24576554-001-04-8::2.5::HTS	0.2507	3.24E-09	8.94E-07
BRD-K61397605-001-03-4::2.5::HTS	0.2501	3.09E-09	9.04E-07
BRD-K36529613-001-02-6::2.5::HTS	0.2473	4.10E-09	1.07E-06
BRD-K68346641-001-01-4::2.5::HTS	0.2470	5.54E-09	1.37E-06
BRD-K00152668-001-01-5::2.5::HTS	0.2458	6.05E-09	1.42E-06
BRD-K15179879-001-03-2::2.5::HTS	0.2448	1.22E-08	2.30E-06
BRD-K58122457-001-02-2::2.5::HTS	0.2438	7.99E-09	1.70E-06
BRD-K73197500-001-02-2::2.5::HTS	0.2417	1.05E-08	2.15E-06
BRD-A72716251-001-01-5::0.91::HTS	0.2410	1.32E-08	2.37E-06
BRD-K04548931-003-16-5::2.5::HTS	0.2407	1.08E-08	2.10E-06
BRD-K69932463-001-10-6::2.5::HTS	0.2384	1.89E-08	3.27E-06
BRD-K66296774-236-11-3::2.63::HTS	0.2359	4.14E-08	6.47E-06
BRD-K92150287-066-01-2::2.5::HTS	0.2342	3.36E-08	5.43E-06
BRD-K43389675-003-20-9::2.5::HTS	0.2336	3.36E-08	5.62E-06
BRD-K56211775-001-03-8::2.5::MTS004	0.2327	6.04E-08	8.85E-06
BRD-A28970875-300-04-9::2.5::HTS	0.2313	7.66E-08	1.06E-05
BRD-K02130563-001-11-4::2.5::HTS	0.2303	5.78E-08	8.73E-06
BRD-K77841042-001-14-1::2.5::HTS	0.2302	6.16E-08	8.75E-06
BRD-K59325863-001-03-6::2.46::HTS	0.2291	1.31E-07	1.66E-05
BRD-K07972848-001-02-0::2.5::HTS	0.2262	8.91E-08	1.19E-05
BRD-A11815689-001-01-1::2.5::HTS	0.2261	9.26E-08	1.20E-05
BRD-K83988098-001-02-0::2.5::HTS	0.2240	1.40E-07	1.68E-05
BRD-K52762805-001-01-2::2.5::HTS	0.2237	1.38E-07	1.70E-05

BRD-K99919177-001-01-3::2.5::MTS004	0.2187	3.58E-07	3.64E-05
BRD-K41312087-001-02-7::2.5::HTS	0.2185	2.50E-07	2.86E-05
BRD-K69694239-001-02-2::2.5::HTS	0.2181	2.33E-07	2.73E-05
BRD-A03506276-001-01-5::2.5::HTS	0.2179	2.71E-07	3.03E-05
BRD-K20463415-300-01-4::2.499966791::MTS004	0.2178	3.99E-07	3.98E-05
BRD-K09426783-300-01-0::2.5::HTS	0.2174	4.49E-07	4.39E-05
BRD-K98795921-001-01-7::2.5::HTS	0.2171	3.24E-07	3.45E-05
BRD-K39484304-001-16-5::2.5::HTS	0.2169	2.86E-07	3.11E-05
BRD-K88510285-001-11-1::2.5::HTS	0.2160	3.26E-07	3.40E-05
BRD-K45293975-001-02-0::2.5::HTS	0.2152	4.97E-07	4.57E-05
BRD-K99475619-001-01-2::2.5::HTS	0.2145	5.47E-07	4.75E-05
BRD-K86687746-001-01-3::2.5::HTS	0.2136	6.08E-07	5.09E-05
BRD-K40331046-305-01-5::2.5::HTS	0.2135	4.51E-07	4.32E-05
BRD-K51967704-001-03-6::2.5::HTS	0.2134	4.79E-07	4.49E-05
BRD-K86525559-001-04-5::2.5::HTS	0.2132	7.54E-07	5.70E-05
BRD-A93236127-001-04-5::2.5::HTS	0.2126	5.27E-07	4.75E-05
BRD-K68904758-001-03-5::2.5::HTS	0.2117	6.36E-07	5.23E-05
BRD-K20605374-001-05-4::2.5::HTS	0.2115	5.41E-07	4.79E-05
BRD-K92657060-001-05-7::2.5::HTS	0.2110	5.74E-07	4.89E-05
BRD-K21680192-300-14-4::2.5::HTS	0.2109	6.99E-07	5.37E-05
BRD-K45724504-001-01-6::1.36::HTS	0.2109	6.57E-07	5.31E-05
BRD-K07955840-001-02-3::2.5::HTS	0.2104	6.81E-07	5.41E-05
BRD-K39171998-001-01-1::2.5::HTS	0.2102	6.96E-07	5.43E-05
BRD-K13050541-001-01-9::2.8::HTS	0.2100	7.83E-07	5.73E-05
BRD-A36057565-001-01-0::2.5::HTS	0.2099	8.33E-07	6.00E-05
BRD-K54247840-004-01-3::2.5::HTS	0.2096	9.86E-07	7.00E-05
BRD-K76703230-004-04-1::2.5::HTS	0.2096	7.71E-07	5.73E-05
BRD-K74193284-001-04-8::2.5::HTS	0.2087	1.60E-06	0.0001
BRD-K41213548-001-02-0::2.5::HTS	0.2083	1.17E-06	8.03E-05
BRD-K01683783-001-02-6::2.5::HTS	0.2067	1.07E-06	7.46E-05
BRD-K22149900-001-03-9::2.371298578::MTS004	0.2064	1.58E-06	0.0001
BRD-K18619710-001-14-4::2.5::HTS	0.2062	1.25E-06	8.26E-05
BRD-K78177893-001-02-4::2.5::HTS	0.2058	1.19E-06	8.07E-05
BRD-K76674262-001-03-3::2.5::HTS	0.2050	1.20E-06	8.06E-05
BRD-K37890730-001-15-1::2.5::HTS	0.2048	1.76E-06	0.0001
BRD-K13390322-001-06-3::2.5::HTS	0.2036	2.02E-06	0.0001
BRD-K30577245-001-04-3::2.5::HTS	0.2015	3.01E-06	0.0002
BRD-K70511574-001-06-9::2.5::HTS	0.2009	2.07E-06	0.0001
BRD-A64064900-001-02-3::2.5::HTS	0.2004	2.95E-06	0.0002
BRD-K58770988-001-01-8::2.5::HTS	0.2002	2.29E-06	0.0001
BRD-K89561498-001-01-7::2.5::HTS	0.1998	2.49E-06	0.0001

BRD-K38852836-001-02-1::2.5::HTS	0.1979	2.80E-06	0.0002
BRD-K49328571-001-15-0::2.5::HTS	0.1975	3.64E-06	0.0002
BRD-A36331462-001-02-1::2.5::HTS	0.1970	3.81E-06	0.0002
BRD-K77638923-001-02-0::2.5::HTS	0.1967	5.20E-06	0.0003
BRD-K82967180-001-01-6::2.500024493::MTS004	0.1957	5.47E-06	0.0003
BRD-K75352575-001-01-3::2.5::HTS	0.1956	3.92E-06	0.0002
BRD-K21025364-001-04-9::2.5::HTS	0.1942	6.82E-06	0.0004
BRD-K43389698-001-09-9::2.4::HTS	0.1941	8.26E-06	0.0004
BRD-K78659596-001-03-9::2.5::HTS	0.1934	5.74E-06	0.0003
BRD-K13646352-001-03-8::2.5::HTS	0.1926	6.05E-06	0.0003
BRD-K96104201-001-01-5::2.5::HTS	0.1919	6.19E-06	0.0003
BRD-K62008436-001-22-1::2.5::HTS	0.1916	6.81E-06	0.0004
BRD-A47953250-001-01-7::2.5::HTS	0.1915	9.21E-06	0.0004
BRD-K96358241-001-01-9::0.94::HTS	0.1910	6.86E-06	0.0003
BRD-K17203476-001-01-9::2.5::HTS	0.1904	6.95E-06	0.0004
BRD-K67844266-003-01-9::2.5::HTS	0.1901	7.14E-06	0.0004
BRD-K22134346-001-24-9::2.42::HTS	0.1899	1.30E-05	0.0006
BRD-K75958547-238-01-0::2.5::HTS	0.1882	8.83E-06	0.0004
BRD-A12417644-001-07-6::2.31::HTS	0.1857	2.02E-05	0.0009
BRD-K68346641-001-02-2::2.5::HTS	0.1844	1.34E-05	0.0006
BRD-K71823332-001-03-7::2.5::HTS	0.1830	1.74E-05	0.0008
BRD-K17061841-001-03-5::2.5::HTS	0.1827	1.57E-05	0.0007
BRD-K53417444-003-03-1::2.5::HTS	0.1823	2.45E-05	0.0011
BRD-K80480517-001-02-4::2.5::HTS	0.1820	1.80E-05	0.0008
BRD-K24666289-001-04-9::2.499988887::MTS004	0.1818	2.46E-05	0.0010
BRD-K10961822-001-05-1::2.5::HTS	0.1816	2.63E-05	0.0011
BRD-K51791723-003-01-7::2.5::HTS	0.1812	1.94E-05	0.0009
BRD-K44227013-001-06-4::2.5::HTS	0.1812	2.02E-05	0.0009
BRD-K49371609-003-03-8::2.5::HTS	0.1811	2.14E-05	0.0009
BRD-K21361524-001-01-1::2.5::HTS	0.1799	2.11E-05	0.0009
BRD-A94756469-001-04-7::2.5::HTS	0.1799	2.12E-05	0.0009
BRD-K03449891-001-08-6::2.5::HTS	0.1795	2.54E-05	0.0010
BRD-K59325863-001-02-8::2.5::HTS	0.1787	2.48E-05	0.0010
BRD-K52233191-001-02-4::2.5::HTS	0.1786	3.18E-05	0.0012
BRD-K13662825-001-07-5::2.5::HTS	0.1785	2.45E-05	0.0010
BRD-K37865504-001-04-1::2.5::HTS	0.1780	2.95E-05	0.0012
BRD-K20958582-001-01-4::2.5::HTS	0.1780	2.96E-05	0.0012
BRD-K72951360-001-01-4::2.5::HTS	0.1775	3.13E-05	0.0012
BRD-A58193911-003-18-7::2.5::HTS	0.1774	4.07E-05	0.0016
BRD-K14658796-003-01-6::2.368766292::MTS004	0.1772	4.01E-05	0.0015

BRD-K28907958-001-04-3::2.5::HTS	0.1767	3.13E-05	0.0012
BRD-K12539581-001-23-7::2.5::HTS	0.1764	3.07E-05	0.0012
BRD-K60230970-001-17-5::2.4::HTS	0.1758	5.81E-05	0.0021
BRD-A36267905-003-18-4::2.5::HTS	0.1745	5.45E-05	0.0020
BRD-K89015388-001-01-7::2.5::HTS	0.1740	4.26E-05	0.0016
BRD-K24187789-001-01-7::2.5::HTS	0.1718	5.18E-05	0.0019
BRD-K54095730-001-03-1::2.5::HTS	0.1712	5.69E-05	0.0021
BRD-K81473043-001-14-6::2.5::HTS	0.1708	9.01E-05	0.0030
BRD-K07881437-001-03-8::2.5::HTS	0.1707	7.86E-05	0.0027
BRD-K22127577-001-03-7::2.58::HTS	0.1705	9.26E-05	0.0030
BRD-K73293050-001-03-1::2.5::HTS	0.1703	6.47E-05	0.0023
BRD-A35588707-001-05-5::2.5::HTS	0.1695	6.65E-05	0.0024
BRD-A50157456-065-20-6::2.5::HTS	0.1693	7.28E-05	0.0025
BRD-K47832606-001-30-1::2.5::HTS	0.1690	7.40E-05	0.0025
BRD-K97746869-001-27-0::2.5::HTS	0.1688	6.68E-05	0.0024
BRD-K87349682-347-03-8::2.36::HTS	0.1687	0.000109984	0.0033
BRD-K11927976-050-02-9::2.5::HTS	0.1687	8.63E-05	0.0028
BRD-K59753975-001-02-6::2.5::MTS004	0.1685	9.36E-05	0.0029
BRD-A27376179-001-01-3::2.5::HTS	0.1682	9.05E-05	0.0029
BRD-K06858286-001-01-3::2.5::HTS	0.1681	7.72E-05	0.0026
BRD-K99545815-001-06-3::2.5::HTS	0.1680	7.27E-05	0.0025
BRD-K69280563-001-01-8::2.5::HTS	0.1676	8.47E-05	0.0029
BRD-A25687296-300-04-3::2.5::HTS	0.1672	8.48E-05	0.0028
BRD-K08542803-001-03-1::0.8::HTS	0.1672	8.49E-05	0.0028
BRD-K33277808-331-01-3::2.5::HTS	0.1664	9.02E-05	0.0029
BRD-K55696337-003-24-4::2.50001813::MTS004	0.1660	0.000119166	0.0036
BRD-K87909389-003-03-4::2.5::HTS	0.1660	9.29E-05	0.0030
BRD-K42973005-001-04-0::2.5::HTS	0.1658	9.33E-05	0.0030
BRD-K93034159-001-25-8::2.5::HTS	0.1656	9.46E-05	0.0030
BRD-K23363278-001-02-1::2.5::HTS	0.1653	9.51E-05	0.0030
BRD-K67977190-066-02-3::2.5::HTS	0.1651	0.0001	0.0036
BRD-K37687095-001-06-9::2.5::HTS	0.1646	0.0001	0.0031
BRD-K81801188-001-02-8::2.5::HTS	0.1644	0.0001	0.0033
BRD-K87696786-003-04-4::2.5::HTS	0.1636	0.0001	0.0037
BRD-K69247067-001-01-8::2.5::HTS	0.1634	0.0001	0.0035
BRD-K22064724-001-01-8::2.499989634::MTS004	0.1633	0.0002	0.0043
BRD-K50859149-001-19-5::2.5::HTS	0.1630	0.0001	0.0037
BRD-K08547377-394-03-5::2.5::HTS	0.1627	0.0002	0.0047
BRD-K54256913-001-08-7::2.5::HTS	0.1621	0.0001	0.0040
BRD-K82135108-001-04-3::2.5::HTS	0.1615	0.0001	0.0041
BRD-K89053832-001-01-0::2.5::HTS	0.1615	0.0001	0.0041

BRD-K56957086-001-06-3::2.5::HTS	0.1614	0.0001	0.0040
BRD-K69776681-001-03-8::2.5::HTS	0.1611	0.0001	0.0042
BRD-K48684885-001-04-2::2.5::HTS	0.1606	0.0002	0.0047
BRD-K24593301-001-02-3::2.54::HTS	0.1602	0.0002	0.0062
BRD-K60446698-001-01-2::2.5::HTS	0.1600	0.0002	0.0047
BRD-A34358106-003-01-6::2.5::HTS	0.1599	0.0002	0.0049
BRD-K05104363-001-10-0::2.5::HTS	0.1599	0.0002	0.0058
BRD-K96253961-046-03-1::2.5::HTS	0.1598	0.0002	0.0047
BRD-K37590257-001-02-8::2.5::HTS	0.1595	0.0002	0.0056
BRD-K88090157-050-10-8::2.5::HTS	0.1588	0.0002	0.0051
BRD-K83837640-001-04-8::2.5::HTS	0.1587	0.0002	0.0063
BRD-K12068470-001-02-5::2.36::HTS	0.1575	0.0003	0.0075
BRD-K47407372-001-17-1::2.5::HTS	0.1572	0.0002	0.0059
BRD-K68164687-001-01-6::2.5::HTS	0.1571	0.0002	0.0058
BRD-K87700323-003-05-1::2.5::HTS	0.1571	0.0002	0.0059
BRD-K64538373-001-01-4::2.5::HTS	0.1568	0.0002	0.0059
BRD-K79821389-001-03-5::2.562009205::MTS004	0.1566	0.0003	0.0071
BRD-K98530306-003-18-3::2.5::HTS	0.1565	0.0002	0.0058
BRD-K61195623-001-01-4::2.5::HTS	0.1564	0.0002	0.0059
BRD-K96631475-001-02-4::2.43::HTS	0.1563	0.0003	0.0082
BRD-K43389675-003-16-7::2.35::HTS	0.1560	0.0004	0.0084
BRD-K06814349-304-01-9::2.5::HTS	0.1556	0.0003	0.0067
BRD-K92093830-003-30-8::2.5::HTS	0.1553	0.0003	0.0065
BRD-K26667523-001-02-5::2.5::HTS	0.1552	0.0003	0.0069
BRD-K13800121-001-01-5::2.5::HTS	0.1548	0.0003	0.0071
BRD-K14560436-001-01-4::2.5::HTS	0.1547	0.0003	0.0068
BRD-K91370081-001-25-1::2.5::HTS	0.1536	0.0003	0.0077
BRD-K81197548-003-01-4::2.5::HTS	0.1531	0.0003	0.0077
BRD-K96233303-001-01-9::2.5::HTS	0.1531	0.0003	0.0076
BRD-K56032964-001-02-1::2.5::HTS	0.1527	0.0003	0.0077
BRD-A68969091-001-12-7::2.57::HTS	0.1523	0.0004	0.0093
BRD-K82181219-001-04-3::2.5::HTS	0.1520	0.0004	0.0084
BRD-K29968218-001-03-2::2.5::HTS	0.1520	0.0004	0.0087
BRD-K12343256-001-08-9::2.5::HTS	0.1519	0.0004	0.0083
BRD-K08248804-001-01-8::2.5::HTS	0.1504	0.0005	0.0117
BRD-K89014967-001-04-3::2.5::HTS	0.1500	0.0004	0.0099
BRD-K56334280-001-05-1::2.5::HTS	0.1494	0.0004	0.0098
BRD-A83644949-236-01-6::2.5::HTS	0.1492	0.0005	0.0117
BRD-A68631409-001-05-4::2.5::HTS	0.1492	0.0005	0.0109
BRD-A91452556-001-04-0::2.5::HTS	0.1489	0.0005	0.0107
BRD-K53414658-001-08-2::2.5::HTS	0.1487	0.0006	0.0129
BRD-K15108141-001-06-6::2.5::HTS	0.1487	0.0006	0.0129

BRD-K43236057-001-07-1::2.6::HTS	0.1486	0.0007	0.0141
BRD-K64866502-001-03-9::2.5::HTS	0.1482	0.0005	0.0117
BRD-K92428232-001-10-6::2.5::HTS	0.1473	0.0005	0.0118
BRD-K55250441-001-13-0::2.5::HTS	0.1472	0.0005	0.0121
BRD-A17846016-001-13-9::2.5::HTS	0.1471	0.0006	0.0122
BRD-K93779381-001-01-9::2.5::HTS	0.1468	0.0005	0.0120
BRD-K78666826-003-01-6::2.5::HTS	0.1466	0.0006	0.0127
BRD-K38548312-001-01-0::2.5::HTS	0.1463	0.0006	0.0126
BRD-K83699324-001-01-1::2.5::HTS	0.1463	0.0007	0.0142
BRD-K15600710-066-04-1::2.5::HTS	0.1462	0.0006	0.0132
BRD-K31086665-005-04-6::2.5::HTS	0.1458	0.0006	0.0130
BRD-K36739687-001-03-0::2.5::HTS	0.1458	0.0006	0.0132
BRD-K21548250-003-19-1::2.5::HTS	0.1454	0.0007	0.0140
BRD-K95573441-001-01-2::2.5::HTS	0.1451	0.0006	0.0137
BRD-K05445342-001-05-3::2.57::HTS	0.1451	0.0009	0.0181
BRD-K72414522-001-06-7::2.5::HTS	0.1444	0.0007	0.0141
BRD-K85178109-001-02-3::2.5::HTS	0.1441	0.0007	0.0148
BRD-K20545304-001-01-6::2.5::HTS	0.1438	0.0008	0.0157
BRD-K99498722-001-02-6::2.5::HTS	0.1432	0.0008	0.0164
BRD-K51318897-001-15-3::2.5::HTS	0.1431	0.0010	0.0190
BRD-K49865102-001-08-4::2.5::HTS	0.1426	0.0009	0.0176
BRD-K21565985-001-22-5::2.5::HTS	0.1423	0.0008	0.0168
BRD-K43621685-001-02-5::2.5::HTS	0.1415	0.0009	0.0181
BRD-K17075857-001-17-6::2.5::HTS	0.1410	0.0009	0.0180
BRD-K09951645-001-06-8::2.5::HTS	0.1409	0.0011	0.0222
BRD-K86797399-001-05-1::2.55::HTS	0.1408	0.0013	0.0239
BRD-K42205652-001-02-7::2.5::HTS	0.1396	0.0010	0.0197
BRD-K62814476-001-03-3::2.5::HTS	0.1396	0.0011	0.0212
BRD-K03384561-001-02-7::2.5::HTS	0.1392	0.0011	0.0212
BRD-K43644456-001-04-0::2.38::HTS	0.1390	0.0015	0.0268
BRD-K23301018-001-11-7::2.63::HTS	0.1390	0.0015	0.0267
BRD-K21782625-001-03-2::2.45::HTS	0.1387	0.0015	0.0268
BRD-K65781196-001-03-0::2.5::HTS	0.1386	0.0012	0.0232
BRD-K45033733-001-12-2::2.5::HTS	0.1381	0.0013	0.0241
BRD-A81513827-001-03-6::2.5::HTS	0.1380	0.0013	0.0240
BRD-K83029223-001-01-3::2.500025378::MTS004	0.1377	0.0015	0.0267
BRD-A03216249-003-24-3::2.5::HTS	0.1376	0.0013	0.0241
BRD-K79254416-001-22-6::2.5::HTS	0.1375	0.0013	0.0240
BRD-K30577245-341-01-9::2.5::HTS	0.1374	0.0013	0.0241
BRD-K26818574-305-07-6::2.5::MTS004	0.1373	0.0015	0.0267
BRD-A47790386-001-03-6::2.61::HTS	0.1370	0.0023	0.0355
BRD-K03601870-001-01-2::2.5::HTS	0.1369	0.0013	0.0244

BRD-K95053546-001-01-9::2.5::HTS	0.1369	0.0013	0.0241
BRD-K59831625-001-01-6::2.5::HTS	0.1366	0.0013	0.0241
BRD-K11973162-003-01-8::2.5::HTS	0.1365	0.0017	0.0289
BRD-K62374002-001-01-5::2.499972663::MTS004	0.1363	0.0016	0.0282
BRD-K57080016-001-15-9::2.5::HTS	0.1363	0.0013	0.0244
BRD-K10843433-001-22-7::2.5::HTS	0.1361	0.0017	0.0291
BRD-K50140147-001-10-1::2.5::HTS	0.1356	0.0015	0.0269
BRD-K94441233-001-13-0::2.6::HTS	0.1355	0.0019	0.0324
BRD-K85140930-001-12-7::2.5::HTS	0.1355	0.0016	0.0287
BRD-K32372024-001-01-0::2.5::HTS	0.1352	0.0021	0.0337
BRD-K10362825-001-03-4::2.5::HTS	0.1352	0.0020	0.0328
BRD-K16189898-003-03-3::2.5::HTS	0.1351	0.0015	0.0267
BRD-K16136380-001-01-7::2.5::HTS	0.1351	0.0020	0.0330
BRD-K73319509-001-08-0::2.5::HTS	0.1350	0.0015	0.0268
BRD-K12329651-001-18-8::2.5::HTS	0.1349	0.0015	0.0266
BRD-A70083328-001-23-5::2.5::HTS	0.1348	0.0016	0.0285
BRD-K55187425-236-05-2::2.5::HTS	0.1345	0.0016	0.0279
BRD-K94436377-001-10-2::2.5::HTS	0.1345	0.0015	0.0270
BRD-K72264770-001-03-2::2.5::MTS004	0.1345	0.0019	0.0317
BRD-A77291778-003-22-0::2.5::HTS	0.1344	0.0016	0.0285
BRD-K81694556-003-01-9::2.5::HTS	0.1343	0.0019	0.0324
BRD-K09443272-001-03-3::2.5::HTS	0.1331	0.0018	0.0305
BRD-K07310275-001-02-5::2.5::HTS	0.1327	0.0018	0.0314
BRD-K40175214-001-11-6::2.5::HTS	0.1325	0.0018	0.0307
BRD-K21025364-001-05-6::2.44::HTS	0.1323	0.0025	0.0384
BRD-K64800655-001-07-4::2.5::HTS	0.1322	0.0018	0.0313
BRD-K56405753-001-02-4::2.5::HTS	0.1321	0.0018	0.0314
BRD-K72703948-001-10-1::2.5::HTS	0.1321	0.0020	0.0331
BRD-K13949769-001-01-7::2.5::HTS	0.1320	0.0019	0.0315
BRD-K68938568-001-01-7::2.5::HTS	0.1319	0.0019	0.0321
BRD-K69650333-003-13-2::2.48::HTS	0.1318	0.0026	0.0393
BRD-K76845197-001-05-4::2.5::HTS	0.1316	0.0020	0.0332
BRD-K92830582-003-10-5::2.5::HTS	0.1315	0.0021	0.0340
BRD-K18324993-001-01-6::2.5::HTS	0.1311	0.0020	0.0333
BRD-K02241333-001-02-7::2.5::HTS	0.1311	0.0021	0.0338
BRD-K71512533-066-02-8::2.5::HTS	0.1311	0.0022	0.0347
BRD-K88061624-001-21-4::2.5::HTS	0.1310	0.0028	0.0417
BRD-K37798499-001-27-2::2.5::HTS	0.1307	0.0022	0.0347
BRD-K04552268-001-02-4::5::HTS	0.1306	0.0026	0.0392
BRD-K82484965-003-03-5::2.5::HTS	0.1306	0.0022	0.0349
BRD-A18992208-003-02-7::2.5::MTS004	0.1304	0.0025	0.0391
BRD-K48173020-001-01-8::2.5::HTS	0.1304	0.0022	0.0351

BRD-K79131256-001-17-9::2.5::HTS	0.1302	0.0022	0.0346
BRD-K87112191-001-04-5::2.5::HTS	0.1295	0.0023	0.0364
BRD-K06234293-001-04-2::2.5::HTS	0.1293	0.0025	0.0389
BRD-K08206212-002-01-0::2.5::HTS	0.1293	0.0028	0.0424
BRD-K50677762-001-01-5::2.5::HTS	0.1292	0.0025	0.0386
BRD-K12251893-065-04-7::2.5::HTS	0.1287	0.0026	0.0394
BRD-K85402309-001-06-6::2.5::HTS	0.1285	0.0026	0.0398
BRD-K28115081-001-02-7::2.5::HTS	0.1285	0.0027	0.0397
BRD-K55305701-001-01-2::2.5::HTS	0.1283	0.0025	0.0387
BRD-K40530731-001-11-6::2.5::HTS	0.1283	0.0040	0.0542
BRD-K02475039-001-01-0::2.5::HTS	0.1283	0.0026	0.0396
BRD-A14344385-001-03-2::2.5::HTS	0.1283	0.0026	0.0395
BRD-K42673188-001-01-1::2.363355746::MTS004	0.1281	0.0031	0.0447
BRD-K64120610-001-01-4::2.5::MTS004	0.1280	0.0031	0.0446
BRD-K76592088-001-02-1::2.5::HTS	0.1280	0.0026	0.0392
BRD-K47079459-004-04-6::2.5::HTS	0.1278	0.0030	0.0440
BRD-K42756753-001-01-9::2.5::HTS	0.1278	0.0032	0.0462
BRD-K82244583-001-01-3::2.5::HTS	0.1278	0.0032	0.0461
BRD-K02594908-001-16-4::2.5::HTS	0.1275	0.0031	0.0448
BRD-K21528677-001-02-8::2.44::HTS	0.1273	0.0036	0.0503
BRD-K19477839-001-07-6::2.5::HTS	0.1272	0.0029	0.0432
BRD-K03063480-001-06-6::2.42::HTS	0.1268	0.0037	0.0516
BRD-K11851476-003-01-7::2.499993171::MTS004	0.1268	0.0034	0.0479
BRD-K63736853-001-04-8::2.5::HTS	0.1267	0.0033	0.0467
BRD-A09062839-003-10-0::2.5::HTS	0.1267	0.0029	0.0426
BRD-A62428732-300-06-3::2.5::HTS	0.1265	0.0029	0.0426
BRD-K12787259-001-04-3::2.5::HTS	0.1263	0.0036	0.0502
BRD-K88568253-011-19-9::2.5::HTS	0.1263	0.0034	0.0480
BRD-A64242993-001-05-6::2.5::HTS	0.1260	0.0031	0.0449
BRD-K20468903-001-01-6::2.49996519::MTS004	0.1256	0.0037	0.0509
BRD-A37492983-001-10-3::2.5::HTS	0.1252	0.0032	0.0461
BRD-K33379087-001-07-5::2.5::HTS	0.1250	0.0035	0.0498
BRD-K23228615-001-02-8::2.5::HTS	0.1249	0.0035	0.0498
BRD-K07790550-001-01-6::2.500001908::MTS004	0.1247	0.0039	0.0536
BRD-A01907367-001-01-7::2.5::HTS	0.1245	0.0035	0.0497
BRD-K04956647-003-02-5::2.5::HTS	0.1241	0.0036	0.0505
BRD-K03243820-001-23-8::2.37::HTS	0.1233	0.0048	0.0624
BRD-K95785537-001-23-1::2.5::HTS	0.1233	0.0041	0.0557
BRD-K32821942-001-21-3::2.5::HTS	0.1232	0.0038	0.0519

BRD-K20168442-004-06-3::2.5::HTS	0.1231	0.0039	0.0536
BRD-K87512222-019-01-7::2.5::HTS	0.1230	0.0038	0.0528
BRD-K04264130-001-01-4::2.5::HTS	0.1230	0.0039	0.0537
BRD-K09711437-001-04-3::2.5::HTS	0.1229	0.0046	0.0608
BRD-K11630072-001-13-2::2.5::HTS	0.1225	0.0051	0.0656
BRD-K46862739-001-14-3::2.499952208::MTS004	0.1224	0.0047	0.0614
BRD-K63784565-001-06-2::2.51::HTS	0.1223	0.0052	0.0664
BRD-K34022604-001-06-6::2.5::HTS	0.1221	0.0041	0.0554
BRD-K79450420-001-02-6::2.5::MTS004	0.1221	0.0048	0.0624
BRD-K01669786-001-02-2::2.5::HTS	0.1218	0.0054	0.0675
BRD-K39120595-304-04-7::2.5::HTS	0.1216	0.0044	0.0583
BRD-A61676498-001-06-6::2.5::HTS	0.1215	0.0043	0.0573
BRD-K47000838-001-01-6::1.04::HTS	0.1215	0.0051	0.0657
BRD-K00259736-001-16-4::2.5::HTS	0.1213	0.0045	0.0604
BRD-A75850590-213-01-0::2.5::HTS	0.1213	0.0053	0.0673
BRD-K92382976-050-01-1::2.5::HTS	0.1211	0.0045	0.0599
BRD-A57798112-001-03-7::2.5::HTS	0.1210	0.0045	0.0605
BRD-A50998626-001-02-1::2.5::HTS	0.1209	0.0046	0.0612
BRD-K63923597-001-10-0::2.5::HTS	0.1208	0.0047	0.0618
BRD-K06426971-001-02-7::2.5::HTS	0.1208	0.0046	0.0607
BRD-K62363391-001-24-9::2.5::HTS	0.1206	0.0047	0.0613
BRD-K80778372-001-02-5::2.5::HTS	0.1197	0.0051	0.0659
BRD-K30237152-300-02-5::2.5::HTS	0.1194	0.0053	0.0675
BRD-K14385366-001-04-4::2.59::HTS	0.1192	0.0067	0.0799
BRD-K95142244-001-01-5::2.5::HTS	0.1190	0.0051	0.0660
BRD-A55312468-001-04-7::1.24::HTS	0.1190	0.0054	0.0675
BRD-K68202742-001-16-5::2.63::HTS	0.1189	0.0066	0.0792
BRD-K87737963-001-06-0::2.5::HTS	0.1187	0.0053	0.0675
BRD-A01636364-003-15-1::2.5::HTS	0.1186	0.0054	0.0678
BRD-K03063480-001-05-8::2.5::HTS	0.1185	0.0052	0.0667
BRD-K18574842-236-17-7::2.5::HTS	0.1185	0.0063	0.0766
BRD-K75360161-001-18-0::2.5::HTS	0.1184	0.0054	0.0680
BRD-K67117832-001-01-9::2.5::MTS004	0.1180	0.0064	0.0778
BRD-K03390685-001-01-7::2.5::HTS	0.1180	0.0058	0.0719
BRD-K43187018-001-03-3::2.52::HTS	0.1176	0.0072	0.0846
BRD-A12077521-003-01-5::2.5::HTS	0.1176	0.0067	0.0802
BRD-K45528773-001-07-2::2.59::HTS	0.1173	0.0073	0.0856
BRD-K13044802-213-20-7::2.5::HTS	0.1172	0.0058	0.0717
BRD-K74227499-001-01-1::2.5::HTS	0.1171	0.0061	0.0751
BRD-K62810658-001-12-2::2.5::HTS	0.1170	0.0061	0.0749
BRD-K73191876-001-11-2::2.5::HTS	0.1170	0.0066	0.0796
BRD-K14235254-001-01-2::2.5::HTS	0.1169	0.0061	0.0750

BRD-K09397065-003-06-1::2.5::HTS	0.1169	0.0070	0.0835
BRD-K74141488-003-11-2::2.5::HTS	0.1168	0.0060	0.0738
BRD-K72815923-034-01-2::2.5::HTS	0.1167	0.0061	0.0750
BRD-A93093700-001-02-9::2.5::HTS	0.1165	0.0061	0.0751
BRD-A83937277-001-10-5::2.5::HTS	0.1163	0.0073	0.0856
BRD-K00634421-001-01-0::2.500042819::MTS004	0.1161	0.0073	0.0857
BRD-A49838158-001-01-1::2.5::HTS	0.1160	0.0065	0.0790
BRD-K26823213-001-02-9::2.5::HTS	0.1158	0.0068	0.0808
BRD-K68405354-001-07-2::2.5::HTS	0.1158	0.0065	0.0787
BRD-K37764012-001-04-1::2.5::HTS	0.1157	0.0065	0.0791
BRD-A29944538-003-08-3::2.5::HTS	0.1152	0.0069	0.0823
BRD-K59647213-001-10-5::2.5::HTS	0.1152	0.0090	0.1007
BRD-K24601397-001-01-1::2.5::HTS	0.1151	0.0071	0.0843
BRD-A13323580-001-04-8::2.5::HTS	0.1149	0.0081	0.0919
BRD-K11636097-001-03-1::2.5::HTS	0.1147	0.0073	0.0855
BRD-K68867920-051-11-5::2.5::HTS	0.1144	0.0072	0.0847
BRD-K83257731-003-23-5::2.5::HTS	0.1142	0.0074	0.0857
BRD-K98684188-001-02-9::2.5::HTS	0.1140	0.0077	0.0885
BRD-K36756879-003-08-7::2.5::HTS	0.1140	0.0076	0.0877
BRD-K67536197-003-11-4::2.5::HTS	0.1138	0.0076	0.0879
BRD-K60025295-003-06-6::2.5::HTS	0.1136	0.0077	0.0887
BRD-K07265709-003-05-6::2.61::HTS	0.1136	0.0094	0.1020
BRD-K81855038-003-13-9::2.5::HTS	0.1135	0.0078	0.0887
BRD-A56085258-001-01-8::2.5::HTS	0.1131	0.0078	0.0892
BRD-K67868012-001-07-6::2.5::HTS	0.1131	0.0077	0.0888
BRD-K81473089-003-26-1::2.5::HTS	0.1130	0.0092	0.1014
BRD-A23124853-001-01-4::2.5::MTS004	0.1130	0.0091	0.1010
BRD-K16485616-001-08-9::2.5::HTS	0.1129	0.0083	0.0937
BRD-K41337261-001-03-0::2.5::HTS	0.1129	0.0078	0.0890
BRD-K27061362-001-01-6::2.499998598::MTS004	0.1126	0.0093	0.1014
BRD-K25970317-001-01-6::2.50000335::MTS004	0.1124	0.0094	0.1018
BRD-K94441233-001-17-1::2.5::HTS	0.1124	0.0092	0.1012
BRD-K65417056-003-10-1::2.5::HTS	0.1123	0.0085	0.0956
BRD-K52559566-001-02-9::2.5::HTS	0.1122	0.0092	0.1009
BRD-K31092604-003-03-5::2.5::HTS	0.1121	0.0088	0.0986
BRD-K06542892-001-01-9::2.5::HTS	0.1118	0.0092	0.1008
BRD-K85307935-236-10-3::2.5::HTS	0.1116	0.0108	0.1133
BRD-K68747584-001-02-0::2.5::HTS	0.1114	0.0089	0.0993
BRD-K88366685-300-06-0::2.5::HTS	0.1110	0.0092	0.1017
BRD-K77251551-304-02-9::2.5::HTS	0.1108	0.0091	0.1012

BRD-K77987382-001-13-2::2.5::HTS	0.1107	0.0092	0.1016
BRD-K19540840-001-09-4::2.5::HTS	0.1106	0.0092	0.1010
BRD-A66435872-332-01-8::2.5::HTS	0.1106	0.0092	0.1008
BRD-K51544265-001-04-2::2.5::HTS	0.1106	0.0104	0.1112
BRD-K89917372-001-01-2::2.5::HTS	0.1105	0.0095	0.1023
BRD-A19795905-001-08-0::2.5::HTS	0.1105	0.0098	0.1050
BRD-K21728777-001-02-3::2.5::HTS	0.1104	0.0096	0.1029
BRD-A72212290-300-01-4::2.5::HTS	0.1104	0.0104	0.1111
BRD-K20482099-001-09-4::2.5::HTS	0.1103	0.0117	0.1205
BRD-K56291712-001-01-0::2.5::HTS	0.1103	0.0094	0.1022
BRD-A46717658-236-02-5::2.5::HTS	0.1102	0.0105	0.1115
BRD-K12539416-001-01-4::2.5::HTS	0.1102	0.0111	0.1158
BRD-K81016934-001-02-0::2.5::HTS	0.1095	0.0116	0.1194
BRD-K78055238-001-01-8::2.5::HTS	0.1092	0.0108	0.1136
BRD-K12079898-001-02-6::2.5::HTS	0.1090	0.0106	0.1121
BRD-K08799216-001-05-3::2.5::HTS	0.1088	0.0121	0.1237
BRD-A42759514-001-20-5::2.5::HTS	0.1088	0.0104	0.1110
BRD-K50853363-001-02-3::2.5::HTS	0.1086	0.0109	0.1142
BRD-K41662846-003-01-5::2.517380705::MTS004	0.1085	0.0123	0.1240
BRD-K16444452-001-09-1::2.5::HTS	0.1084	0.0108	0.1136
BRD-K61250484-001-02-3::2.5::HTS	0.1081	0.0112	0.1169
BRD-A57382968-001-29-0::2.5::HTS	0.1079	0.0111	0.1157
BRD-K46625559-001-01-8::2.50003993::MTS004	0.1077	0.0128	0.1275
BRD-A67862938-034-14-9::2.5::HTS	0.1077	0.0115	0.1187
BRD-K72280606-001-01-2::2.499993939::MTS004	0.1075	0.0130	0.1286
BRD-A00055058-001-01-0::2.325889319::MTS004	0.1075	0.0130	0.1283
BRD-K31627533-001-09-5::2.5::HTS	0.1075	0.0118	0.1217
BRD-K07881437-001-04-6::2.41::HTS	0.1074	0.0141	0.1367
BRD-K64890080-001-13-8::2.5::HTS	0.1072	0.0124	0.1248
BRD-K84810405-003-01-1::2.5::HTS	0.1070	0.0138	0.1354
BRD-K18834913-001-01-1::2.5::HTS	0.1070	0.0120	0.1229
BRD-K95901403-001-04-5::2.5::HTS	0.1068	0.0121	0.1237
BRD-K12762134-001-06-2::2.5::HTS	0.1067	0.0127	0.1267
BRD-K00535541-001-05-5::2.5::HTS	0.1066	0.0140	0.1363
BRD-K40255344-001-17-6::2.5::HTS	0.1064	0.0122	0.1241
BRD-K85751432-001-03-3::2.5::HTS	0.1063	0.0137	0.1341
BRD-K23677682-003-01-2::2.5::HTS	0.1062	0.0124	0.1248
BRD-K78118466-001-03-3::2.5::HTS	0.1062	0.0124	0.1251
BRD-K12184916-001-15-4::2.5::HTS	0.1061	0.0127	0.1267

BRD-K96550715-001-02-6::2.5::HTS	0.1060	0.0128	0.1269
BRD-K17294426-050-12-3::2.5::HTS	0.1059	0.0126	0.1268
BRD-K82603084-408-01-1::2.5::HTS	0.1059	0.0140	0.1363
BRD-A25004090-001-08-4::2.5::HTS	0.1058	0.0131	0.1289
BRD-K23779958-001-05-8::2.5::HTS	0.1054	0.0160	0.1490
BRD-K20655524-003-12-0::2.5::HTS	0.1053	0.0152	0.1443
BRD-K81795818-019-01-4::2.5::HTS	0.1053	0.0146	0.1399
BRD-A48300215-001-04-8::2.5::HTS	0.1051	0.0148	0.1408
BRD-K76534306-001-22-7::2.5::HTS	0.1048	0.0140	0.1366
BRD-K66956375-001-11-3::2.5::HTS	0.1046	0.0140	0.1363
BRD-K64888243-001-03-7::2.5::HTS	0.1045	0.0141	0.1364
BRD-A87715314-003-14-4::2.5::HTS	0.1044	0.0148	0.1408
BRD-K27737647-300-03-0::2.5::HTS	0.1044	0.0147	0.1408
BRD-K58148589-001-03-6::2.5::HTS	0.1043	0.0145	0.1390
BRD-K98493452-001-14-9::2.5::HTS	0.1040	0.0144	0.1388
BRD-K02764365-001-13-6::2.5::HTS	0.1039	0.0145	0.1392
BRD-K06502269-001-02-6::2.5::MTS004	0.1038	0.0165	0.1513
BRD-K53156626-001-01-3::2.5::MTS004	0.1034	0.0169	0.1545
BRD-K34157611-001-16-0::2.5::HTS	0.1033	0.0160	0.1489
BRD-K41160163-001-06-8::2.5::HTS	0.1033	0.0153	0.1439
BRD-K06557128-001-07-0::2.5::HTS	0.1032	0.0156	0.1469
BRD-K92000912-001-12-6::2.5::HTS	0.1032	0.0156	0.1467
BRD-K32501161-300-06-2::2.5::HTS	0.1031	0.0162	0.1494
BRD-K48247567-001-01-9::2.5::HTS	0.1029	0.0160	0.1493
BRD-K44004064-001-07-3::2.5::HTS	0.1029	0.0162	0.1494
BRD-K86887724-001-10-6::2.59::HTS	0.1029	0.0178	0.1610
BRD-K35626314-003-01-1::2.499986242::MTS004	0.1028	0.0175	0.1590
BRD-K86972824-001-01-4::2.5::HTS	0.1027	0.0180	0.1616
BRD-K90168339-001-08-6::2.5::HTS	0.1027	0.0160	0.1487
BRD-K93123848-001-04-1::2.5::HTS	0.1026	0.0159	0.1492
BRD-K59875992-001-01-8::2.5::HTS	0.1023	0.0160	0.1485
BRD-K06519765-001-01-1::2.5::MTS004	0.1023	0.0181	0.1627
BRD-K28217197-001-01-4::2.5::HTS	0.1021	0.0162	0.1496
BRD-K82103381-003-09-4::2.500073689::MTS004	0.1021	0.0184	0.1638
BRD-K27799744-001-02-8::2.52::HTS	0.1020	0.0198	0.1715
BRD-K77554836-001-11-6::2.57::HTS	0.1020	0.0198	0.1716
BRD-K49313711-001-05-4::2.5::HTS	0.1018	0.0170	0.1545
BRD-A12896037-001-04-3::2.5::HTS	0.1017	0.0191	0.1688
BRD-K92303087-001-01-6::2.5::HTS	0.1015	0.0169	0.1543
BRD-K17016787-001-16-7::2.5::HTS	0.1015	0.0171	0.1555
BRD-K25494650-003-01-	0.1014	0.0192	0.1695

8::2.660853131::MTS004			
BRD-K35240538-001-26-2::2.5::HTS	0.1013	0.0197	0.1716
BRD-K49372556-001-04-1::2.5::MTS004	0.1011	0.0195	0.1703
BRD-K49197823-001-09-8::2.5::HTS	0.1011	0.0182	0.1630
BRD-K09619578-001-01-3::2.5::HTS	0.1009	0.0178	0.1613
BRD-K50055394-001-03-6::2.701086832::MTS004	0.1009	0.0197	0.1713
BRD-K31698212-001-02-9::2.5::HTS	0.1007	0.0178	0.1611
BRD-K11590034-002-03-4::2.49::HTS	0.1007	0.0214	0.1820
BRD-A55424491-001-19-9::2.5::HTS	0.1002	0.0193	0.1691
BRD-K49962337-001-02-9::2.56::HTS	0.1002	0.0220	0.1853
BRD-K02407574-001-09-7::2.5::HTS	0.1000	0.0186	0.1655
BRD-K65814004-003-02-9::2.5::HTS	0.0999	0.0196	0.1707
BRD-K05181084-003-03-7::2.5::HTS	0.0998	0.0193	0.1694
BRD-K45114938-236-01-8::2.35::HTS	0.0997	0.0231	0.1896
BRD-K33453211-003-11-5::2.5::HTS	0.0997	0.0192	0.1692
BRD-K17295893-001-01-0::2.499987615::MTS004	0.0996	0.0215	0.1823
BRD-A28301258-001-03-0::2.5::HTS	0.0996	0.0200	0.1723
BRD-K18157228-001-01-7::2.5::HTS	0.0995	0.0194	0.1697
BRD-A79981887-003-16-4::2.5::HTS	0.0995	0.0192	0.1695
BRD-K58550667-001-08-7::2.5::HTS	0.0993	0.0201	0.1733
BRD-K92984783-003-05-7::2.5::HTS	0.0993	0.0197	0.1714
BRD-K06980535-003-25-9::2.5::HTS	0.0992	0.0222	0.1863
BRD-K85146014-314-05-4::2.5::HTS	0.0991	0.0238	0.1928
BRD-A58947127-001-01-6::2.5::MTS004	0.0990	0.0223	0.1864
BRD-A50764878-003-02-8::2.5::HTS	0.0990	0.0204	0.1747
BRD-K12513978-001-24-1::2.5::HTS	0.0988	0.0228	0.1887
BRD-K32842773-434-01-8::2.5::MTS004	0.0987	0.0226	0.1874
BRD-K79132813-001-09-0::2.5::HTS	0.0986	0.0211	0.1803
BRD-A89082344-001-08-7::2.500026693::MTS004	0.0986	0.0229	0.1889
BRD-K54472332-001-03-4::2.5::HTS	0.0986	0.0204	0.1747
BRD-K50163129-001-01-4::2.5::HTS	0.0985	0.0213	0.1814
BRD-K59369769-001-20-3::2.5::HTS	0.0984	0.0206	0.1761
BRD-K04701033-001-04-7::2.499987566::MTS004	0.0982	0.0233	0.1895
BRD-A48261811-001-08-1::2.5::HTS	0.0981	0.0238	0.1929
BRD-K13261168-300-01-0::2.5::HTS	0.0980	0.0217	0.1836
BRD-K56691760-001-01-2::2.5::HTS	0.0980	0.0217	0.1836
BRD-K62736196-015-11-0::2.41::HTS	0.0979	0.0253	0.2010
BRD-A65248799-001-01-7::2.5::HTS	0.0979	0.0214	0.1818
BRD-K47869605-001-32-0::2.5::HTS	0.0978	0.0233	0.1899

BRD-A51829654-003-07-7::2.5::HTS	0.0978	0.0219	0.1848
BRD-K31468676-237-01-8::2.5::HTS	0.0978	0.0244	0.1968
BRD-K49338325-001-03-4::2.5::HTS	0.0976	0.0236	0.1916
BRD-K36258877-001-03-1::2.5::HTS	0.0975	0.0224	0.1869
BRD-A07000685-001-04-4::2.5::HTS	0.0974	0.0226	0.1874
BRD-A79803969-003-16-1::2.5::HTS	0.0973	0.0230	0.1895
BRD-K80043866-236-01-1::2.5::HTS	0.0970	0.0224	0.1870
BRD-A41250306-001-11-7::2.5::HTS	0.0970	0.0225	0.1873
BRD-A11990600-001-03-4::2.5::HTS	0.0968	0.0258	0.2031
BRD-K19203487-001-01-4::2.5::HTS	0.0968	0.0231	0.1898
BRD-A26711594-003-10-6::2.5::HTS	0.0966	0.0231	0.1895
BRD-K59317601-001-05-5::2.5::HTS	0.0965	0.0232	0.1898
BRD-K63919159-003-07-0::2.5::HTS	0.0963	0.0261	0.2046
BRD-K35329391-334-01-7::2.5::HTS	0.0960	0.0244	0.1967
BRD-K42500029-001-03-5::2.5::HTS	0.0958	0.0245	0.1969
BRD-A48015106-004-08-9::2.5::HTS	0.0956	0.0244	0.1964
BRD-K20431737-001-08-1::2.5::HTS	0.0956	0.0251	0.2002
BRD-A13598753-001-01-2::2.5::HTS	0.0954	0.0252	0.2008
BRD-A09554849-236-07-6::2.5::HTS	0.0953	0.0256	0.2023
BRD-A64227845-004-09-3::2.5::HTS	0.0952	0.0250	0.2002
BRD-A94323479-001-02-8::2.5::HTS	0.0952	0.0251	0.2003
BRD-A84687895-001-03-2::2.5::HTS	0.0952	0.0257	0.2029
BRD-A16700644-003-04-4::2.5::HTS	0.0951	0.0258	0.2035
BRD-K49555808-001-03-9::2.5::HTS	0.0951	0.0274	0.2119
BRD-K63068307-001-08-9::2.5::HTS	0.0951	0.0263	0.2059
BRD-K34185671-001-02-8::2.5::HTS	0.0951	0.0264	0.2060
BRD-K42066335-001-01-0::2.499995517::MTS004	0.0950	0.0281	0.2145
BRD-A87479750-001-02-7::2.5::HTS	0.0950	0.0260	0.2038
BRD-K91601245-001-12-0::2.5::HTS	0.0950	0.0277	0.2136
BRD-K08310154-001-03-8::2.5::HTS	0.0949	0.0267	0.2079
BRD-M29936662-001-02-0::2.5::HTS	0.0946	0.0259	0.2039
BRD-K12852738-001-01-2::2.584725941::MTS004	0.0946	0.0288	0.2175
BRD-K88677950-001-03-9::2.5::HTS	0.0944	0.0269	0.2092
BRD-K70401845-003-09-6::2.5::HTS	0.0944	0.0267	0.2077
BRD-K04923131-001-15-4::2.5::HTS	0.0943	0.0279	0.2134
BRD-K32847234-065-04-6::2.5::HTS	0.0943	0.0279	0.2135
BRD-K07220430-001-18-4::2.5::HTS	0.0943	0.0273	0.2111
BRD-A66116161-001-02-3::2.5::HTS	0.0942	0.0277	0.2131
BRD-K04111260-001-10-0::2.5::HTS	0.0942	0.0278	0.2130
BRD-K85119730-001-28-9::2.5::HTS	0.0940	0.0293	0.2200
BRD-K12867552-001-04-7::2.5::HTS	0.0939	0.0306	0.2259

BRD-K51333959-003-01-3::2.5::HTS	0.0935	0.0277	0.2134
BRD-K36055864-001-19-2::2.5::HTS	0.0934	0.0285	0.2167
BRD-A05729358-001-02-0::2.5::HTS	0.0934	0.0288	0.2174
BRD-A45543382-001-10-0::2.5::HTS	0.0933	0.0292	0.2196
BRD-A89175223-051-14-8::2.5::HTS	0.0932	0.0285	0.2164
BRD-K21782625-001-02-4::2.5::HTS	0.0932	0.0319	0.2305
BRD-K11452913-001-01-9::2.5000112::MTS004	0.0931	0.0316	0.2299
BRD-K81616657-001-01-4::2.665005855::MTS004	0.0930	0.0317	0.2304
BRD-K82960980-003-01-9::2.5::HTS	0.0929	0.0294	0.2197
BRD-A34255068-001-34-5::2.5::HTS	0.0929	0.0300	0.2229
BRD-K92241597-001-06-0::2.5::HTS	0.0928	0.0290	0.2185
BRD-U08520523-000-01-0::2.5::HTS	0.0927	0.0355	0.2450
BRD-A86216746-046-02-6::2.5::HTS	0.0925	0.0324	0.2336
BRD-K72462751-001-02-7::2.5::HTS	0.0924	0.0297	0.2219
BRD-K28143534-001-02-2::2.5::HTS	0.0923	0.0334	0.2367
BRD-K36207157-001-09-6::2.5::HTS	0.0923	0.0302	0.2243
BRD-K76239644-001-01-8::2.5::HTS	0.0923	0.0304	0.2250
BRD-K60585088-001-01-5::2.5::HTS	0.0922	0.0304	0.2251
BRD-A46335897-003-27-8::2.499949994::MTS004	0.0921	0.0333	0.2368
BRD-A45153512-001-01-0::2.5::HTS	0.0921	0.0330	0.2362
BRD-K42348709-003-13-1::2.5::HTS	0.0920	0.0328	0.2360
BRD-K40715924-001-01-1::2.5::HTS	0.0920	0.0311	0.2291
BRD-K59524082-001-01-3::2.5::HTS	0.0920	0.0307	0.2263
BRD-K74501079-001-18-1::2.5::HTS	0.0920	0.0320	0.2312
BRD-K42221274-003-07-7::2.5::HTS	0.0919	0.0318	0.2306
BRD-A10523515-001-07-9::2.5::HTS	0.0918	0.0316	0.2300
BRD-K99879819-001-02-1::2.5187366::MTS004	0.0917	0.0341	0.2399
BRD-K25875056-001-02-0::2.5::HTS	0.0917	0.0316	0.2303
BRD-K03273112-001-01-8::2.5::HTS	0.0917	0.0314	0.2302
BRD-K58486055-001-02-7::2.5::HTS	0.0917	0.0313	0.2299
BRD-K68488863-001-04-9::2.5::HTS	0.0916	0.0314	0.2305
BRD-A43882281-001-15-5::2.5::HTS	0.0915	0.0339	0.2388
BRD-K34068325-066-03-7::2.499970684::MTS004	0.0915	0.0346	0.2425
BRD-K06357403-001-01-4::2.49996939::MTS004	0.0914	0.0348	0.2429
BRD-K92073408-001-16-5::2.5::HTS	0.0914	0.0319	0.2307
BRD-K83064458-001-23-0::2.5::HTS	0.0912	0.0329	0.2358
BRD-A28467416-002-01-0::2.5::HTS	0.0911	0.0346	0.2423

BRD-K84937637-001-09-9::2.5::HTS	0.0911	0.0375	0.2516
BRD-K06878038-001-18-6::2.5::HTS	0.0911	0.0334	0.2372
BRD-K91758890-001-10-0::2.5::HTS	0.0910	0.0331	0.2369
BRD-K81405859-300-01-4::2.5::MTS004	0.0909	0.0358	0.2461
BRD-K40302533-001-02-9::2.5::HTS	0.0909	0.0328	0.2361
BRD-K86003836-001-10-5::2.5::HTS	0.0907	0.0334	0.2368
BRD-K35520305-001-17-7::2.5::HTS	0.0906	0.0346	0.2421
BRD-A07932845-050-15-8::2.5::HTS	0.0906	0.0332	0.2371
BRD-K20285085-074-04-5::2.5::HTS	0.0905	0.0387	0.2535
BRD-A08545410-311-03-4::2.5::HTS	0.0905	0.0387	0.2532
BRD-K92723993-001-06-7::2.5::HTS	0.0905	0.0332	0.2369
BRD-K06335600-003-20-7::2.5::HTS	0.0905	0.0341	0.2398
BRD-K10598093-303-06-1::2.5::HTS	0.0905	0.0428	0.2651
BRD-K35629949-001-02-0::2.5::HTS	0.0904	0.0352	0.2437
BRD-A02710418-003-11-8::2.5::HTS	0.0901	0.0353	0.2440
BRD-A18056626-003-22-3::2.5::HTS	0.0901	0.0348	0.2426
BRD-K38323065-001-19-9::2.5::HTS	0.0900	0.0351	0.2436
BRD-K73753850-003-01-6::2.499957259::MTS004	0.0899	0.0379	0.2521
BRD-K57275767-001-03-2::2.5::HTS	0.0899	0.0358	0.2459
BRD-K33732501-004-01-0::2.5::HTS	0.0897	0.0364	0.2491
BRD-K26531771-001-02-2::2.35::HTS	0.0897	0.0405	0.2574
BRD-K44067360-001-30-3::2.5::HTS	0.0897	0.0389	0.2530
BRD-K13810148-311-03-2::2.5::HTS	0.0897	0.0349	0.2424
BRD-K59456551-001-22-6::2.51::HTS	0.0897	0.0406	0.2577
BRD-A09349126-001-10-7::2.5::HTS	0.0897	0.0391	0.2531
BRD-K28352084-001-02-3::2.5::HTS	0.0896	0.0358	0.2457
BRD-K32107296-001-16-9::2.5::HTS	0.0894	0.0367	0.2495
BRD-K57545991-050-23-1::2.5::HTS	0.0894	0.0355	0.2451
BRD-K38868394-001-01-3::2.5::HTS	0.0893	0.0383	0.2527
BRD-K01493881-001-29-4::2.5::HTS	0.0893	0.0373	0.2517
BRD-K78318619-001-02-7::2.5::HTS	0.0893	0.0356	0.2451
BRD-K15014948-001-10-3::2.500062642::MTS004	0.0893	0.0392	0.2527
BRD-A96407378-001-15-1::2.5::HTS	0.0893	0.0366	0.2495
BRD-K36965586-003-08-8::2.5::HTS	0.0891	0.0367	0.2497
BRD-K97808269-001-02-7::2.5::HTS	0.0891	0.0374	0.2513
BRD-K19615002-001-02-3::2.5::HTS	0.0891	0.0369	0.2501
BRD-K45117373-001-02-9::2.5::HTS	0.0891	0.0388	0.2535
BRD-K66241279-001-01-8::2.5::HTS	0.0890	0.0363	0.2485
BRD-A29485665-001-12-8::2.5::HTS	0.0889	0.0378	0.2522
BRD-K25394294-001-14-9::2.5::HTS	0.0889	0.0364	0.2487
BRD-A95121829-001-08-9::2.5::HTS	0.0889	0.0378	0.2520

BRD-K83766205-001-03-8::2.5::HTS	0.0889	0.0376	0.2520
BRD-K61951118-001-07-9::2.5::HTS	0.0888	0.0368	0.2499
BRD-K88646909-004-16-2::2.5::HTS	0.0887	0.0378	0.2525
BRD-K91544578-001-03-8::2.5::HTS	0.0886	0.0374	0.2516
BRD-K93231391-300-05-6::2.5::HTS	0.0885	0.0372	0.2522
BRD-K47376733-001-08-1::2.5::HTS	0.0884	0.0380	0.2516
BRD-A22844106-001-26-0::2.5::HTS	0.0883	0.0386	0.2535
BRD-K34713073-001-01-3::2.5::HTS	0.0883	0.0381	0.2519
BRD-K79595931-312-01-3::2.5::HTS	0.0883	0.0396	0.2543
BRD-K49554218-001-01-0::2.5::HTS	0.0883	0.0396	0.2545
BRD-K27182532-001-02-3::2.5::HTS	0.0882	0.0379	0.2518
BRD-K35559145-050-08-5::2.5::HTS	0.0882	0.0380	0.2514
BRD-K53734668-003-02-0::2.66::HTS	0.0882	0.0423	0.2630
BRD-A49035384-003-28-9::2.39::HTS	0.0881	0.0442	0.2716
BRD-K42436189-001-01-2::2.5::HTS	0.0881	0.0382	0.2521
BRD-A08187463-001-12-9::2.5::HTS	0.0881	0.0425	0.2635
BRD-K48722833-001-07-6::2.5::HTS	0.0881	0.0391	0.2534
BRD-K71075609-003-01-0::2.5::HTS	0.0881	0.0386	0.2536
BRD-K47557313-001-02-7::2.5::HTS	0.0881	0.0390	0.2535
BRD-K41599323-001-02-3::2.5::HTS	0.0880	0.0388	0.2535
BRD-K19761926-001-02-8::2.5::HTS	0.0880	0.0399	0.2553
BRD-K15898725-001-01-7::2.5::HTS	0.0880	0.0391	0.2535
BRD-K86109159-001-02-6::2.5::HTS	0.0878	0.0388	0.2532
BRD-A76672327-003-02-0::2.5::HTS	0.0877	0.0420	0.2621
BRD-K96194081-001-10-2::2.5::HTS	0.0877	0.0391	0.2528
BRD-K47598052-001-15-8::2.5::HTS	0.0876	0.0417	0.2612
BRD-K91456750-238-01-7::2.5::HTS	0.0876	0.0394	0.2538
BRD-K10196357-003-02-0::2.5::HTS	0.0876	0.0424	0.2635
BRD-K51313569-003-03-3::2.5::HTS	0.0875	0.0404	0.2571
BRD-K31313613-003-04-2::2.5::HTS	0.0874	0.0407	0.2573
BRD-A00578795-001-04-3::2.5::HTS	0.0874	0.0399	0.2553
BRD-A25569250-001-03-3::2.5::HTS	0.0873	0.0415	0.2609
BRD-K02464583-001-02-2::2.5::HTS	0.0873	0.0401	0.2560
BRD-K34672903-001-01-7::2.5::HTS	0.0872	0.0407	0.2575
BRD-K35189033-001-26-1::2.5::HTS	0.0872	0.0406	0.2576
BRD-K25433859-003-25-4::2.5::HTS	0.0871	0.0456	0.2743
BRD-A67516570-001-02-8::2.5::HTS	0.0871	0.0413	0.2603
BRD-K48195801-001-01-6::2.5::HTS	0.0871	0.0437	0.2690
BRD-K86882815-001-03-2::2.5::HTS	0.0870	0.0451	0.2750
BRD-K71221037-001-01-6::2.5::HTS	0.0869	0.0413	0.2604
BRD-K83834509-001-01-1::2.5::HTS	0.0869	0.0415	0.2605
BRD-K90563805-001-02-6::2.5::MTS004	0.0867	0.0451	0.2746
BRD-A59174698-003-18-5::2.5::HTS	0.0867	0.0417	0.2609

BRD-K89272762-001-12-7::2.5::HTS	0.0867	0.0414	0.2602
BRD-K12102668-001-25-5::2.5::HTS	0.0867	0.0421	0.2625
BRD-K12609457-001-03-1::2.5::HTS	0.0866	0.0462	0.2753
BRD-A96485169-001-14-3::2.5::HTS	0.0865	0.0420	0.2622
BRD-K53814070-310-01-3::2.5::HTS	0.0864	0.0454	0.2741
BRD-K61536336-001-01-7::2.5::HTS	0.0862	0.0431	0.2662
BRD-A67101513-001-03-7::2.5::HTS	0.0861	0.0435	0.2684
BRD-K81645907-001-01-1::2.5::HTS	0.0861	0.0493	0.2865
BRD-K22520627-001-01-7::2.5::HTS	0.0860	0.0447	0.2743
BRD-K30550578-001-01-3::2.5::HTS	0.0859	0.0449	0.2749
BRD-K49659468-436-01-2::2.500002467::MTS004	0.0859	0.0474	0.2796
BRD-K59332007-300-02-7::2.5::HTS	0.0858	0.0435	0.2683
BRD-A49765801-001-04-1::2.5::HTS	0.0856	0.0459	0.2747
BRD-K34415467-003-13-0::2.5::HTS	0.0856	0.0451	0.2749
BRD-A92800748-001-05-5::2.5::HTS	0.0855	0.0454	0.2742
BRD-K64881305-001-03-7::2.5::HTS	0.0855	0.0452	0.2746
BRD-K37561857-001-15-5::2.5::HTS	0.0854	0.0476	0.2804
BRD-K82142815-001-19-1::2.5::HTS	0.0854	0.0453	0.2744
BRD-K07940445-310-02-9::2.5::HTS	0.0854	0.0452	0.2744
BRD-K88405679-003-03-1::2.5::HTS	0.0853	0.0458	0.2747
BRD-A75726477-003-21-3::2.5::HTS	0.0853	0.0449	0.2749
BRD-A92439610-001-05-7::2.5::HTS	0.0853	0.0453	0.2741
BRD-K42191735-001-05-3::2.5::HTS	0.0849	0.0457	0.2746
BRD-K59036917-001-16-7::2.5::HTS	0.0849	0.0457	0.2744
BRD-A75817871-001-06-7::2.5::HTS	0.0849	0.0474	0.2798
BRD-K75608666-001-08-3::2.5::HTS	0.0849	0.0492	0.2867
BRD-K52075715-001-06-7::2.5::HTS	0.0848	0.0461	0.2750
BRD-A19195498-050-14-1::2.5::HTS	0.0848	0.0463	0.2754
BRD-K37714784-305-02-1::2.5::HTS	0.0848	0.0465	0.2761
BRD-K47978074-001-02-4::2.5::HTS	0.0847	0.0496	0.2878
BRD-K55675242-001-03-0::2.5::HTS	0.0847	0.0496	0.2876
BRD-A18611368-001-01-5::2.5::HTS	0.0847	0.0474	0.2794
BRD-K41438959-001-01-7::2.5::HTS	0.0847	0.0464	0.2758
BRD-A10111329-001-04-6::2.5::HTS	0.0845	0.0479	0.2811
BRD-K97530723-001-20-9::2.5::HTS	0.0844	0.0471	0.2786
BRD-A72767275-003-01-6::2.5::HTS	0.0844	0.0487	0.2845
BRD-K49111258-003-29-7::2.5::HTS	0.0844	0.0486	0.2839
BRD-K22261391-001-01-2::2.5::HTS	0.0841	0.0478	0.2811
BRD-K88172511-003-21-1::2.5::HTS	0.0839	0.0495	0.2873
BRD-K96259238-001-01-4::2.5::HTS	0.0838	0.0492	0.2865
BRD-K93258693-001-17-3::2.5::HTS	-0.0835	0.0500	0.2892
BRD-K71926323-001-13-8::2.5::HTS	-0.0841	0.0485	0.2836

BRD-A34751532-001-06-9::2.5::HTS	-0.0844	0.0484	0.2840
BRD-K79145628-001-05-5::2.5::HTS	-0.0848	0.0467	0.2767
BRD-A94008949-001-01-9::2.5::HTS	-0.0851	0.0460	0.2751
BRD-K97101532-001-02-8::2.5::HTS	-0.0852	0.0451	0.2753
BRD-K19061412-001-02-4::2.5::HTS	-0.0854	0.0461	0.2748
BRD-A72401848-001-02-2::2.551316459::MTS004	-0.0855	0.0484	0.2837
BRD-K48329353-001-01-3::2.5::HTS	-0.0857	0.0447	0.2745
BRD-K54262262-001-09-0::2.5::HTS	-0.0859	0.0455	0.2740
BRD-K48061146-066-01-7::2.5::HTS	-0.0863	0.0455	0.2742
BRD-K03739921-001-06-8::2.5::HTS	-0.0875	0.0409	0.2582
BRD-K22503835-001-11-0::2.5::HTS	-0.0877	0.0395	0.2545
BRD-K72339976-065-04-7::2.5::HTS	-0.0880	0.0403	0.2570
BRD-K79584249-001-01-3::2.5::HTS	-0.0880	0.0398	0.2550
BRD-K53319039-001-01-8::2.5::MTS004	-0.0882	0.0425	0.2635
BRD-K88186167-001-04-8::2.5::HTS	-0.0883	0.0377	0.2522
BRD-K24476327-001-01-3::2.50002878::MTS004	-0.0897	0.0384	0.2524
BRD-K77300776-001-02-5::2.5::HTS	-0.0898	0.0373	0.2516
BRD-K35367061-001-01-1::2.5::MTS004	-0.0899	0.0378	0.2517
BRD-A74667430-001-23-6::2.5::HTS	-0.0902	0.0338	0.2384
BRD-K82118441-001-03-7::2.646883368::MTS004	-0.0903	0.0373	0.2520
BRD-K09126848-003-01-9::2.5::HTS	-0.0904	0.0348	0.2430
BRD-K08188887-001-02-4::2.5::HTS	-0.0910	0.0336	0.2377
BRD-K27393415-001-01-0::2.5::HTS	-0.0913	0.0333	0.2369
BRD-K98174813-001-18-0::2.5::HTS	-0.0918	0.0315	0.2306
BRD-K92467929-001-01-1::2.5::HTS	-0.0920	0.0304	0.2248
BRD-K21160401-065-01-0::2.5::HTS	-0.0924	0.0297	0.2222
BRD-K77677632-003-02-6::2.5::HTS	-0.0931	0.0299	0.2225
BRD-K73477617-001-08-5::2.5::HTS	-0.0934	0.0286	0.2162
BRD-K11663430-001-02-3::2.5::HTS	-0.0934	0.0284	0.2159
BRD-K20714604-003-02-9::2.5::HTS	-0.0938	0.0277	0.2134
BRD-K37723606-001-01-5::2.5::HTS	-0.0940	0.0281	0.2148
BRD-K56735750-001-06-8::2.5::HTS	-0.0943	0.0272	0.2113
BRD-K26024145-001-01-9::2.5::MTS004	-0.0945	0.0290	0.2186
BRD-K61688984-001-02-9::2.5::HTS	-0.0956	0.0250	0.2005
BRD-K23925186-001-01-0::2.5::HTS	-0.0956	0.0255	0.2020
BRD-K70177501-003-01-4::2.5::HTS	-0.0967	0.0239	0.1932
BRD-K85066592-001-01-6::2.5::HTS	-0.0978	0.0229	0.1891
BRD-K88560311-011-06-3::2.5::HTS	-0.0980	0.0221	0.1856
BRD-K52618540-001-09-9::2.5::HTS	-0.0985	0.0233	0.1896
BRD-K56301217-001-07-4::2.5::HTS	-0.0989	0.0200	0.1722

BRD-K89714990-001-01-5::2.5::HTS	-0.1001	0.0189	0.1676
BRD-A43435802-001-02-0::2.5::HTS	-0.1003	0.0183	0.1632
BRD-K21673112-003-01-3::2.5::HTS	-0.1003	0.0190	0.1691
BRD-K02389548-001-02-5::2.5::HTS	-0.1012	0.0179	0.1612
BRD-K87535339-001-01-6::2.53::HTS	-0.1027	0.0168	0.1539
BRD-K31111078-001-04-2::2.5::HTS	-0.1040	0.0152	0.1441
BRD-K83186168-001-01-8::2.5::HTS	-0.1044	0.0157	0.1474
BRD-K24821794-001-01-4::2.5::HTS	-0.1046	0.0142	0.1369
BRD-K57774929-001-01-3::2.643351543::MTS004	-0.1054	0.0148	0.1411
BRD-K48578705-001-17-6::2.5::HTS	-0.1056	0.0149	0.1417
BRD-K43449605-001-01-0::2.5::HTS	-0.1056	0.0136	0.1336
BRD-A37441042-003-04-8::2.5::HTS	-0.1062	0.0127	0.1268
BRD-A78877355-001-03-0::2.5::HTS	-0.1068	0.0119	0.1220
BRD-K29458283-001-29-9::2.5::HTS	-0.1073	0.0122	0.1238
BRD-K08111240-001-02-8::2.5::HTS	-0.1082	0.0114	0.1186
BRD-A30655177-001-14-0::2.5::HTS	-0.1100	0.0101	0.1085
BRD-K79095980-001-09-6::2.5::HTS	-0.1105	0.0093	0.1014
BRD-K66808046-065-03-7::2.5::HTS	-0.1110	0.0105	0.1115
BRD-K41876534-001-13-7::2.5::HTS	-0.1141	0.0075	0.0865
BRD-K18135438-001-14-2::2.5::MTS004	-0.1142	0.0083	0.0941
BRD-A29289453-001-04-7::2.5::HTS	-0.1190	0.0052	0.0663
BRD-K68188368-001-09-4::2.5::HTS	-0.1222	0.0041	0.0558
BRD-K89085489-001-23-2::2.499967871::MTS004	-0.1237	0.0042	0.0568
BRD-K25140590-001-03-0::2.5::HTS	-0.1242	0.0035	0.0497
BRD-K70402238-001-02-8::2.5::HTS	-0.1289	0.0024	0.0381
BRD-K15179513-001-03-4::2.5::HTS	-0.1297	0.0024	0.0378
BRD-K15164005-001-05-6::2.5::HTS	-0.1312	0.0022	0.0347
BRD-K28537285-001-01-8::2.499968715::MTS004	-0.1323	0.0022	0.0349
BRD-K13314656-001-01-0::2.5::HTS	-0.1377	0.0014	0.0260
BRD-K98203492-003-04-1::2.5::HTS	-0.1388	0.0013	0.0249
BRD-K24790130-001-07-1::2.5::HTS	-0.1409	0.0009	0.0185
BRD-K82928847-001-04-7::2.5::HTS	-0.1413	0.0010	0.0202
BRD-K76180386-001-01-6::2.5::HTS	-0.1427	0.0008	0.0163
BRD-K61868322-050-01-7::2.5::HTS	-0.1433	0.0008	0.0157
BRD-K72726508-001-02-1::2.5::HTS	-0.1559	0.0003	0.0066
BRD-K02265150-001-26-5::2.5::HTS	-0.1592	0.0002	0.0052
BRD-K86358349-001-02-0::2.440473496::MTS004	-0.1661	0.0001	0.0036
BRD-K95523387-001-09-6::2.5::HTS	-0.1723	4.89E-05	0.0018
BRD-K52313696-001-12-3::2.5::HTS	-0.1726	6.57E-05	0.0024

BRD-K34533029-001-08-5::2.5::HTS	-0.1796	2.51E-05	0.0010
