# Supplementary figures and images for: ATAXIC: An Algorithm to Quantify Transcriptomic Perturbation Heterogeneity in Single Cancer Cells
Source: J Oncol. 2022 Aug 31;2022:4106736. doi: 10.1155/2022/4106736 (PMC9452944; doi:10.1155/2022/4106736)

Fig. S1

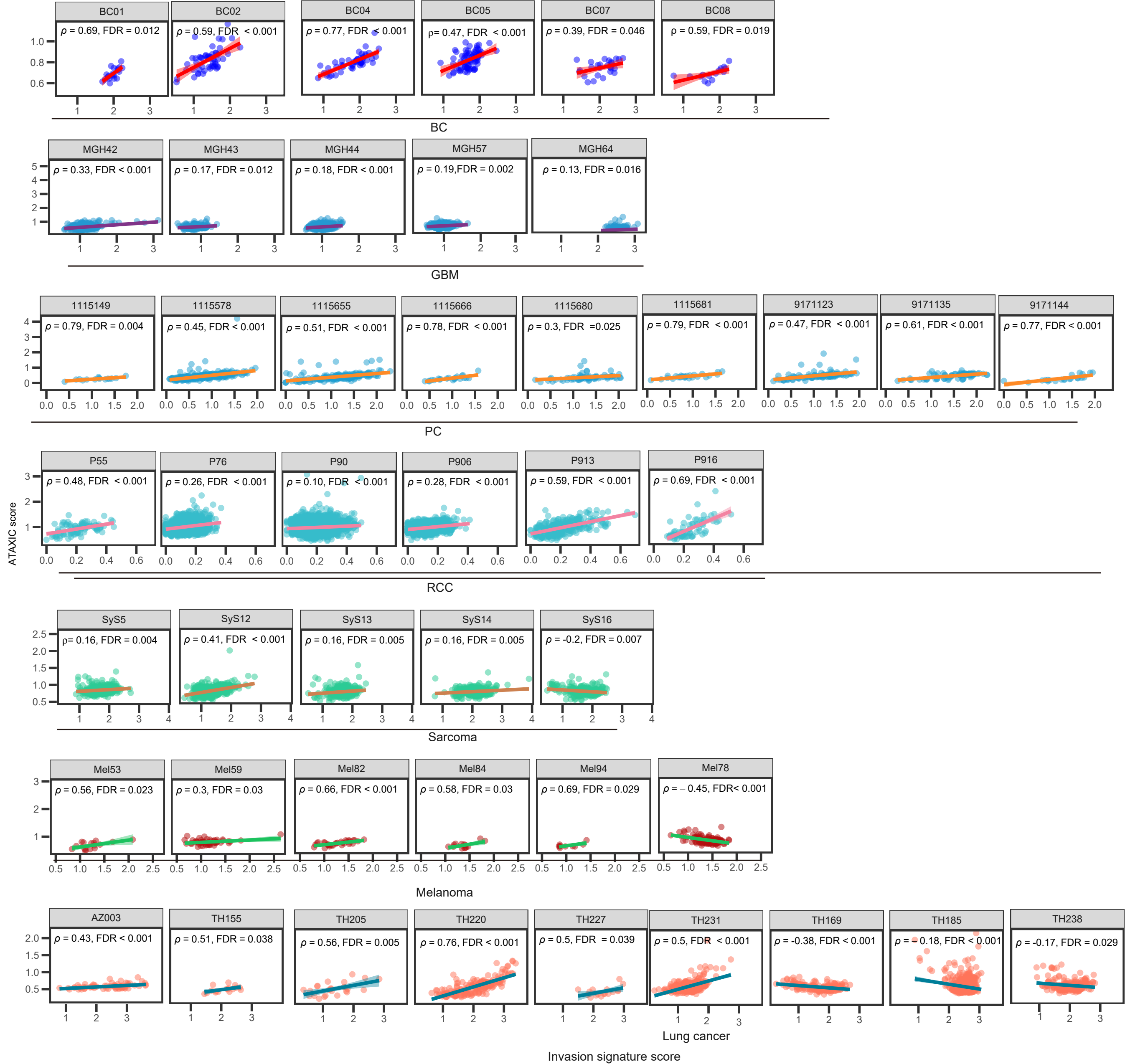

Supplement: Supplementary Materials — Table S1: The numbers of patients and their cancer single cells in each cancer type. Table S2: The marker or pathway gene sets of the signatures and pathways analyzed in this study. Table S3: Significant correlations between the viability values and ATAXIC scores in the 578 cancer cell lines for 728 compounds. The Spearman correlation coefficients, P values, and adjusted P values (FDR) are shown. Figure S1 Spearman correlations between ATAXIC scores and the enrichment scores of the invasion signature in single cells from breast cancer (BC), glioma (GBM), prostate cancer (PC), renal cell carcinoma (RCC), sarcoma, melanoma, and lung cancer. The Spearman correlation coefficients and adjusted P values (FDR) are shown. Figure S2: Correlations of ATAXIC scores with oncogenic pathways in cancer. Spearman correlations between ATAXIC scores and the enrichment scores of the TGF-β (A), Wnt (B), JAK-STAT (C), PI3K-Akt (D), Notch (E), and Hedgehog signaling pathways (F) in single cells from individual patients of eight cancer types. The Spearman correlation coefficients and adjusted P values (FDR) are shown. [file 4106736.f1.zip › figureS1.pdf]

Fig .S2

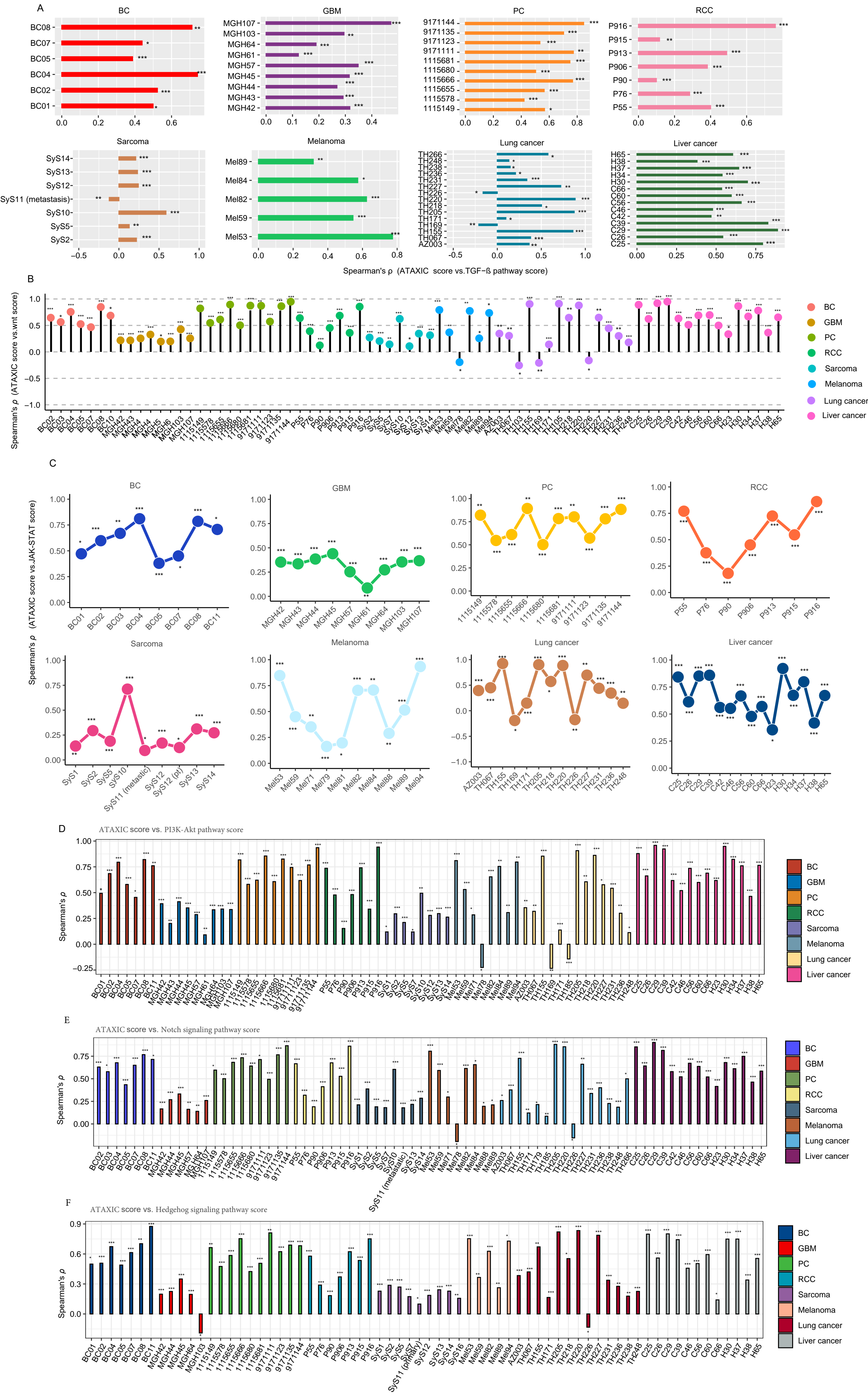

Supplement: Supplementary Materials — Table S1: The numbers of patients and their cancer single cells in each cancer type. Table S2: The marker or pathway gene sets of the signatures and pathways analyzed in this study. Table S3: Significant correlations between the viability values and ATAXIC scores in the 578 cancer cell lines for 728 compounds. The Spearman correlation coefficients, P values, and adjusted P values (FDR) are shown. Figure S1 Spearman correlations between ATAXIC scores and the enrichment scores of the invasion signature in single cells from breast cancer (BC), glioma (GBM), prostate cancer (PC), renal cell carcinoma (RCC), sarcoma, melanoma, and lung cancer. The Spearman correlation coefficients and adjusted P values (FDR) are shown. Figure S2: Correlations of ATAXIC scores with oncogenic pathways in cancer. Spearman correlations between ATAXIC scores and the enrichment scores of the TGF-β (A), Wnt (B), JAK-STAT (C), PI3K-Akt (D), Notch (E), and Hedgehog signaling pathways (F) in single cells from individual patients of eight cancer types. The Spearman correlation coefficients and adjusted P values (FDR) are shown. [file 4106736.f1.zip › figureS2.pdf]
